# Modular Hydrogel Vaccine for Programmable and Coordinate Elicitation of Cancer Immunotherapy

**DOI:** 10.1002/advs.202301789

**Published:** 2023-05-24

**Authors:** Panpan Ji, Wenqi Sun, Siyan Zhang, Yuqi Xing, Chen Wang, Mengying Wei, Qiuyun Li, Gang Ji, Guodong Yang

**Affiliations:** ^1^ Department of Digestive Surgery Xijing Hospital Fourth Military Medical University Shaanxi 710032 China; ^2^ Department of Ultrasound Diagnostics Tangdu Hospital Fourth Military Medical University Shaanxi 710038 China; ^3^ The State Laboratory of Cancer Biology Department of Biochemistry and Molecular Biology Fourth Military Medical University Shaanxi 710032 China; ^4^ Department of Breast Surgery The Affiliated Tumor Hospital of Guangxi Medical University Nanning Guangxi 530021 China

**Keywords:** antigen presentation, cancer immunotherapy, exosomes, hydrogel, programmed release, vaccine

## Abstract

Immunotherapy holds great promise for the treatment of malignant cancer. However, the lack of sufficient tumor neoantigens and incomplete dendritic cell (DC) maturation compromise the efficacy of immunotherapy. Here, a modular hydrogel‐based vaccine capable of eliciting a powerful and sustained immune response is developed. Briefly, CCL21a and Exo^GM‐CSF+Ce6^ (tumor cell‐derived exosomes with granulocyte‐macrophage colony‐stimulating factor (GM‐CSF) mRNA encapsulated inside and sonosensitizer chlorin e6 (Ce6) incorporated in the surface) are mixed with nanoclay and gelatin methacryloyl, forming the hydrogel designated as CCL21a/Exo^GM‐CSF+Ce6^@nanoGel. The engineered hydrogel releases CCL21a and GM‐CSF with a time gap. The earlier released CCL21a diverts the tumor‐draining lymph node (TdLN) metastatic tumor cells to the hydrogel. Consequently, the trapped tumor cells in the hydrogel, in turn, engulf the Ce6‐containing exosomes and thus are eradicated by sonodynamic therapy (SDT), serving as the antigen source. Later, together with the remnant CCL21a, GM‐CSF produced by cells engulfing Exo^GM‐CSF+Ce6^ continuously recruits and provokes DCs. With the two programmed modules, the engineered modular hydrogel vaccine efficiently inhibits tumor growth and metastasis via diverting TdLN metastatic cancer to hydrogel, killing the trapped tumor cells, and eliciting prolonged and powerful immunotherapy in an orchestrated manner. The strategy would open an avenue for cancer immunotherapy.

## Graphical abstract

The designed CCL21a/Exo^GM‐CSF+Ce6^@nanoGel hydrogel releases CCL21a and granulocyte‐macrophage colony‐stimulating factor in distinct profiles. In combination with sonodynamic therapy, the modular hydrogel vaccine system efficiently inhibits tumor growth and metastasis via diverting tumor‐draining lymph node metastatic cancer to hydrogel, killing the trapped tumor cells, and eliciting prolonged and powerful immunotherapy in an orchestrated manner.

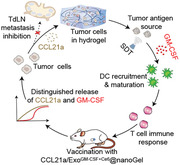

## 1 Introduction

Immunotherapy has emerged as an effective and exciting strategy to elicit systemic antitumor responses, both for hematologic and solid malignancy.^[^ ^1^ ^]^ Besides immune checkpoint blockade therapy and chimeric antigen receptor T‐cell therapy, therapeutic cancer vaccines have also been intensively studied in the past decade.^[^ ^2^ ^]^ However, the efficacy of therapeutic cancer vaccines remains limited.^[^ ^3^ ^]^

Both the immunosuppressive microenvironment and lack of optimal vaccine design are the root causes of compromised efficacy.^[^ ^4^ ^]^ For example, dendritic cells (DCs) are essential for priming and activating effector T cells.^[^ ^5^ ^]^ However, tumor cells are found to suppress DC function with different means or directly recruit immune‐suppressive DCs via remodeling the tumor microenvironment.^[^ ^6^ ^]^ Bypassing the immune suppressive microenvironment would boost the vaccine efficacy. On the other hand, huge progress has also been made with respect to antigen sources and delivery systems for antigen/adjuvants. Tumor cell lysates, peptides, and nucleic acids carrying/expressing the tumor‐associated antigens are explored as the antigen sources.^[^ ^7^ ^]^ LNPs, virus vectors, and even modified autologous DCs are designed to deliver the antigen/adjuvants to elicit desirable and long‐lasting immune responses.^[^ ^8^ ^]^ However, novel strategies with a rational capacity to revert immunosuppression, deliver antigen/adjuvants effectively, and generate long‐lasting effects remain badly needed.

Recently, hydrogels emerge as potent tumor vaccine delivery systems for their high biocompatibility, large loading capacity, and tailorable release behaviors.^[^ ^9^ ^]^ For example, gelatin methacryloyl (GelMA)‐based hydrogels have the essential properties of native extracellular matrix (ECM), which allow cells to proliferate and spread in the gel. Moreover, hybrid hydrogel systems can be developed by mixing GelMA with nanoparticles, such as nanoclay, graphene oxide, and other polymers, for specific biological applications.^[^ ^10^ ^]^ Exosomes, which are nanostructured membrane vesicles released by the cells when multivesicular bodies fuse with the plasma membrane, are considered as a new cell‐free tumor vaccine when originally carry the antigen or are engineered to deliver tumor antigen.^[^ ^11^ ^]^ Exosomes could be also engineered to load mRNAs, which could be efficiently translated into protein when endocytosed by the recipient cells.^[^ ^12^ ^]^

Theoretically, recruiting cancer cells to the hydrogel, inducing cancer cell immunogenic cell death (ICD) and activating antigen presentation in a sequential manner, would be a promising strategy, though it is technically challenged. To achieve the goal, two different cytokines recruiting cancer cells and DCs, respectively, should be selected and engineered to release in distinct profiles. CCL21a is a chemokine that promotes the growth and metastasis of many tumor types, including melanomas, breast, thyroid, colon, head, and neck cancers, via pairing with CCR7 expressed on tumor cells.^[^ ^13^ ^]^ In addition, forced expression of CCL21a is also found to elicit potent antitumor immunity in clinical trials.^[^ ^14^ ^]^ It is thus reasonable to assume that a higher concentration of CCL21a released from the hydrogel could divert TdLN metastatic cancer to the hydrogel. GM‐CSF is generally considered a potent cytokine in homing and activating antigen presentation cells. GM‐CSF‐based vaccines have been shown to elicit potent antitumors in preclinical experiments via upregulation of co‐stimulatory (CD80 and CD86) and MHC class II molecules.^[^ ^15^ ^]^ To achieve a distinct release profile of CCL21a and GM‐CSF from the gel, CCL21a could be directly embedded in the hydrogel while GM‐CSF mRNA could be loaded into exosomes, which in turn soaked in the hydrogel. To this end, GM‐CSF could be released in a later period when the exosomes are endocytosed by the recruited cancer cells. Second, cancer cells should be locally eradicated in the hydrogel. In sonodynamic therapy, ultrasound (US) irradiation exerts a local and efficient anti‐tumor effect when Chlorin e6 (Ce6) accumulates in tumor tissues. Ce6‐loaded nanoparticles are widely used to increase the local drug concentration of Ce6 in tumor cells, as tumor cells are efficient in the uptake of nanoparticles.^[^ ^16^ ^]^ mRNA is encapsulated inside the exosomes, while hydrophobic molecules are incorporated into the membrane. Thus, GM‐CSF mRNA and Ce6 could be simultaneously loaded into the same exosomes. Together, we could see the combination of CCL21a, GM‐CSF, exosomes, and Ce6 will recruit cancer cells, induce ICD, and activate antigen presentation in a coordinated manner.

**Scheme 1:**
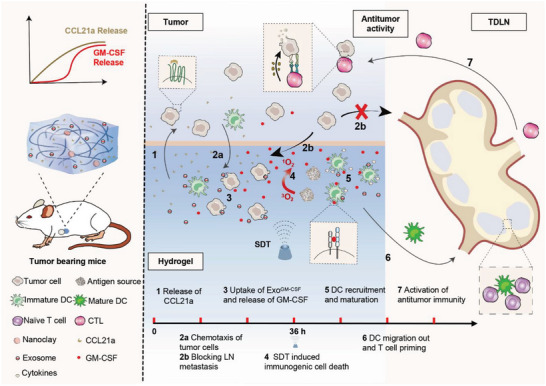
CCL21a, Exo^GM‐CSF+Ce6^ (tumor cell‐derived exosomes with GM‐CSF mRNA encapsulated inside and sonosensitizer Ce6 incorporated in the surface), and GelMA and Nanoclay are mixed to form the modular hydrogel, namely CCL21a/Exo^GM‐CSF+Ce6^@nanoGel. After gelation beside the inoculated tumor, the modular hydrogel releases CCL21a and GM‐CSF with a time gap. The early released CCL21a diverts the tumor‐draining lymph node (TdLN) metastatic tumor cells to the hydrogel. Consequently, the trapped tumor cells in the hydrogel, in turn, engulf the Ce6‐containing exosomes and thus are eradicated by sonodynamic therapy (SDT), serving as the antigen source. Later, the multifunctional Exo^GM‐CSF+Ce6^, together with the remnant CCL21a, continuously recruits and provokes DCs. With the two modules, the hydrogel vaccine system efficiently inhibits tumor growth and metastasis via diverting TdLN metastatic cancer to hydrogel, killing the trapped tumor cells, and eliciting prolonged and powerful immunotherapy in a programmed manner.

In this study, we have developed a hydrogel vaccine nodule favorable for eliciting a powerful and sustained immune response. The chemokine CCL21a and engineered exosome Exo^GM‐CSF+Ce6^ are mixed with GelMA and nanoclay to generate the modular hydrogel, namely CCL21a/Exo^GM‐CSF+Ce6^@nanoGel (**Scheme** **1**). The Exo^GM‐CSF+Ce6^ is tumor cell‐derived exosomes additionally engineered with GM‐CSF mRNA encapsulated inside and Ce6 incorporated in the surface. With the two modules in the hydrogel, the hydrogel vaccine could divert TdLN metastatic cancer to hydrogel, kill trapped cancer cells, and elicit prolonged and powerful immunotherapy in a programmable and orchestrated manner (Scheme 1), maximizing the efficacy of immunotherapy.

## 2 Results

### 2.1 Encapsulation and Control Release of CCL21a from the Composited Hydrogel

**Figure 1:**
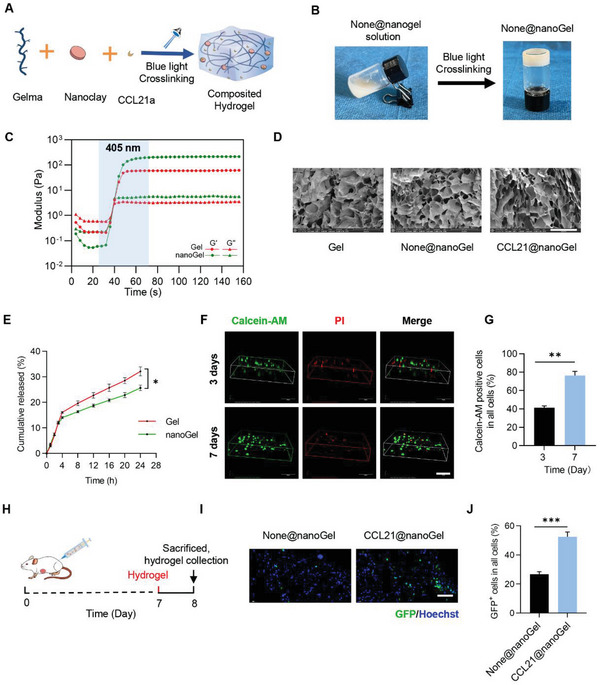
Preparation and characterization of CCL21a@nanoGel. A) Schematic showing the formulation of CCL21a@nanoGel. B) Representative image showing the sol–gel transition of None@nanogel. C) Rheological analysis of the Gel and None@nanoGel. The curves of the energy storage modulus (G′) and loss modulus (G″) during the crosslinking of Gel (10 wt%) or None@nanoGel (10 wt%). D) Scanning electron microscope images of Gel (GelMA), None@nanoGel (Nanoclay+GelMA), and CCL21a@nanoGel (CCL21a+Nanoclay+GelMA). Scale bars, 300 µm. E) Profile of CCL21a released from the indicated hydrogels. Data are expressed as mean ± S.E.M (Standard Error of Mean) of three independent experiments. *, *p* < 0.05 by two‐way ANOVA. F) Cell viability of CT26.WT in the hydrogels was measured by Calcein‐AM/PI staining at the indicated time. Calcein‐AM, green; PI, red. Scale bar, 100 µm. G) The hydrogels were treated with gel lysis solution at 37 °C and isolated Calcein‐AM positive cells were counted. Data are expressed as mean ± S.E.M of three independent experiments. **p < 0.01 by the unpaired Student's *t*‐test. H) Schematic illustration of the experimental procedure. CT26.WT‐GFP colon tumor‐bearing mice were injected with None@nanoGel or CCL21a@nanoGel beside the tumor on day 7, followed by the analysis of the gel on day 8. I) Representative fluorescence microscopic images showing the chemotaxis of CT26.WT‐GFP. Scale bar, 100 µm. J) The hydrogels were treated with gel lysis solution at 37 °C and the isolated GFP^+^ cancer cells were then counted. Data are expressed as mean ± S.E.M of three independent experiments. ***p < 0.001 by the unpaired Student's *t*‐test.

To induce chemotaxis of CCR7^+^ tumor cells, CCL21a was added into the GelMA or GelMA+nanoclay solution, followed by gelation using light radiation (405 nm) (**Figure** **1**A). As expected, light radiation induced a sol–gel transition (Figure 1B). To further explore the hydrogel properties, the gelation process was observed and recorded by rheometer at 37 °C. The elastic modulus (*G*′) and viscous modulus (*G*″) were very low before gelation. During gelation, *G*′ increased faster than *G*″ over time in both control Gel and None@nanoGel. In addition, both *G*′ and *G*″ were higher in None@nanoGel, in comparison with those in control Gel (Figure 1C). Both Gel and None@nanoGel degraded in a slow and steady manner. The addition of nanoclay obviously slowed the degradation rate (Figure S1, Supporting Information).

Scanning electron microscopy (SEM) analysis further confirmed the porous network structure of the formed hydrogel. No significant differences in the structure were observed among the hydrogels, composed of GelMA (Gel), GelMA+nanoclay (None@nanoGel), and CCL21a+ GelMA+nanoclay (CCL21a@nanoGel) (Figure 1D). Next, we explored the degradation ratio of different samples. CCL21a was released in a sustained manner from both CCL21a@Gel and CCL21a@nanoGel, with a slower speed in the latter gel (Figure 1E), which may be attributed to the chain entanglements caused by undissolved nanoclay.

Biocompatibility is of vital importance for materials applied in the biomedical field. To confirm the biocompatibility of the hydrogel, CT26.WT cells were cultured in the hydrogel CCL21a@nanoGel. The live/dead assay showed that the majority of the CT26.WT cells were stained green (live cells), with only a few cells stained red (dead cells) (Figure 1F). Moreover, CT26.WT cells grew well in the hydrogels, as seen from the increased viable cells 7 days after culture (Figure 1G). Similarly, CCL21a@nanoGel had profound biocompatibility with DC2.4, a DC cell line (Figure S2, Supporting Information). All these data suggested that composited CCL21a@nanoGel had excellent biocompatibility for cell culture.

We next observed the chemotactic effects of CCL21a@nanoGel on CT26.WT‐GFP in vivo. Mice inoculated with CT26.WT‐GFP cells were injected with CCL21a@nanoGel, with None@nanoGel serving as a control (Figure S3, Supporting Information), and 8 days later, the mice were sacrificed and hydrogels were harvested for confocal fluorescence microscopy analysis (Figure 1H). There were more GFP^+^ cancer cells observed in CCL21a@nanoGel than that in None@nanoGel (Figure 1I). The hydrogels were treated with gel lysis solution at 37 °C and the cells in the gel were then isolated and counted, which further confirmed that the ratio of GFP^+^ cancer cells was much higher in CCL21a@nanoGel (Figure 1J). Together, these results confirmed that CCL21a@nanoGel had a higher capacity to recruit CT26.WT‐GFP cells possibly via CCL21a/CCR7 axis.

### 2.2 Preparation and Characterization of Exo^GM‐CSF^

**Figure 2:**
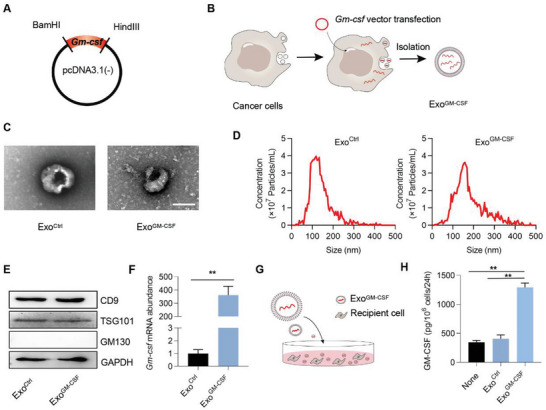
Preparation and characterization of Exo^GM‐CSF^. A) Cloning of GM‐CSF expressing plasmid. The CDS of GM‐CSF was cloned into the plasmid with restricted endonuclease sites as indicated. B) Schematic illustration of GM‐CSF mRNA encapsulation into the exosomes. The donor cells were forced to express GM‐CSF upon transfection of the plasmid. The high level of GM‐CSF was thus passively enriched in the exosomes. C) Transmission electron microscopy (TEM) analysis of Exo^GM‐CSF^. Scale bar, 100 nm. D) Size distribution of the indicated exosomes was analyzed by NanoSight. E) Western blotting analysis of the surface markers of Exo^Ctrl^ and Exo^GM‐CSF^. GAPDH served as the loading control. Representative data of three different experiments. F) GM‐CSF mRNA abundance in exosomes derived from recipient cells treated as indicated. Data are expressed as mean ± S.E.M of three independent experiments. **p < 0.01 by the unpaired Student's *t*‐test. G) Schematic illustration of the exosome‐mediated GM‐CSF mRNA delivery into the recipient cells, where the mRNA is translated into the functional protein. 2 × 10^6^ recipient cells were treated with 40 µg Exo^Ctrl^ or Exo^GM‐CSF^, and 24 h later cell‐free supernatants were collected for analysis of the level of GM‐CSF. H) ELISA analysis of GM‐CSF in the supernatant of recipient cells. Data are expressed as mean ± S.E.M of three independent experiments. **, *p* < 0.01 by one‐way ANOVA.

To achieve potent and prolonged DC activation, Exo^GM‐CSF^, in which GM‐CSF mRNA was loaded into the exosomes, was designed and soaked in the hydrogel. Theoretically, when the exosomes are engulfed by the cells, GM‐CSF protein would be produced in a durable manner. The plasmid expressing GM‐CSF was thus constructed (**Figure** **2**A and Figure S4, Supporting Information) and transfected into exosome donor cells (Figure 2B). As exosomes are also a source of antigen,^[^ ^17^ ^]^ we thus selected cancer cells to be inoculated as the donor cells. The derived exosomes were thus denoted as Exo^GM‐CSF^, while the control exosomes from cells transfected with the control empty vector were denoted as Exo^Ctrl^. Both Exo^Ctrl^ and Exo^GM‐CSF^ had similar cup‐ or round‐shaped morphology with a size from 50 to 150 nm (Figure 2C,D). Western blotting indicated that these exosomes were positive for inclusive surface markers, including TSG101 and CD9, and the absence of exclusive markers GM130, confirming the identity of exosomes (Figure 2E). As expected, high expression of GM‐CSF mRNA in donor cells (Figure S5, Supporting Information) was passively packaged into the exosomes, with a more than 300‐fold increase of the mRNA copies in Exo^GM‐CSF^ (Figure 2F). In order to further evaluate whether Exo^GM‐CSF^ could transfer the mRNA to the recipient cells and in turn produce GM‐CSF protein, recipient cells were treated with Exo^GM‐CSF^, the control Exo^None^ (Exosomes from cancer cells with no additional treatment), or Exo^Ctrl^, and 24 h later cell‐free supernatants were collected for analysis of the level of GM‐CSF using enzyme‐linked immunosorbent assay (ELISA) assay (Figure 2G). Compared to the control groups, the level of GM‐CSF was much higher in the Exo^GM‐CSF^‐treated group (Figure 2H).

### 2.3 CCL21a/Exo^GM‐CSF+Ce6^@nanoGel in Combination with SDT Induces Cancer Cell Death

**Figure 3:**
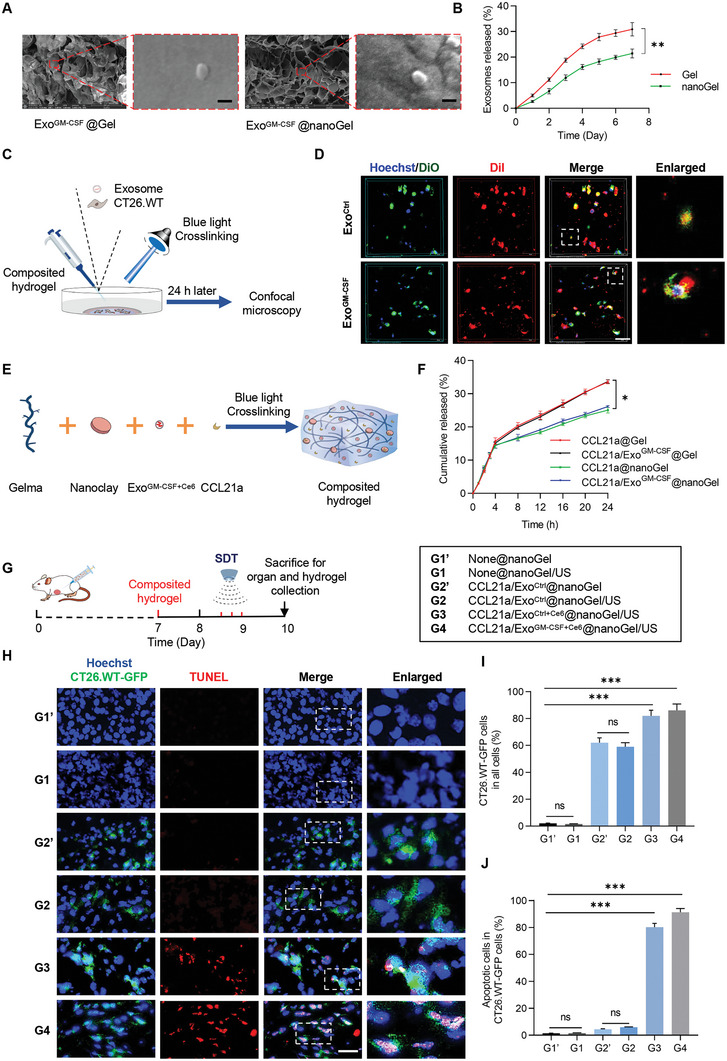
CCL21a/Exo^GM‐CSF+Ce6^@nanoGel in combination with SDT induces cancer cell death. A) Scanning electron micrographs of Exo^GM‐CSF^@Gel and Exo^GM‐CSF^@nanoGel. Scale bars, 100 nm. B) Releasing profile of Exo^GM‐CSF^ from the hydrogels. Data are expressed as mean ± S.E.M of three independent experiments. **, P ＜0.01 by two‐way ANOVA. C) Schematic showing the experimental procedure of tracking exosome engulfed by the cells in the hydrogel. D) The intracellular distribution of DiI‐labeled exosomes in hydrogels was analyzed by fluorescence microscopy. Nuclei were counterstained with Hoechst. Cell membranes were labeled with DiO. Scale bar, 50 µm. E) Schematic showing composited hydrogel formation. F) Profile of CCL21a released from the indicated hydrogels. Data are expressed as mean ± S.E.M of three independent experiments. *, *p* < 0.05 by two‐way ANOVA. G) Schematic illustration of sonodynamic therapy effect in vivo. CT26.WT‐GFP tumor‐bearing mice were randomly divided into six groups: None@nanoGel (G1′), None@nanoGel/US group (G1), CCL21a/Exo^Ctrl^@nanoGel (G2′), CCL21a/Exo^Ctrl^@nanoGel/US group (G2), CCL21a/ Exo^Ctrl+Ce6^ @nanoGel/US group (G3), and CCL21a/Exo^GM‐CSF+Ce6^@nanoGel/US group (G4). On the 7th day of subcutaneous tumor inoculation, hydrogels with different components were injected beside the tumor. Ultrasound irradiation was performed every 4 h for three times on day 8. The mice were sacrificed and hydrogels were harvested for further analysis on day 10. H) Representative images of TUNEL staining of hydrogel slices from different groups. Nuclei, blue; CT26.WT‐GFP, Green; TUNEL, red. Scale bar, 30 µm. I) Percentages of CT26.WT‐GFP in the hydrogel on day 10. Data are expressed as mean ±S.E.M of three independent experiments. ***, *p* < 0.001 by one‐way ANOVA. J) Percentage of apoptotic CT26.WT‐GFP cells in indicated groups. Data are expressed as mean ± S.E.M of three independent experiments. ***, *p* < 0.001; ns, no significance by one‐way ANOVA.

We next loaded the exosomes into the hydrogel by mixing the exosomes with the gel solution. Briefly, 1 × 10^9^/mL exosomes were added into Gel or nanoGel solution and gelation was obtained after crosslinking by blue light radiation for 30 s. SEM analysis revealed that the addition of exosomes had no significant effect on the morphology (**Figure** **3**A). A large number of exosomes was observed on the surface of the composite hydrogels (Figure 3A). The exosomes released profiles from hydrogels were measured by qPCR analysis of the *Gapdh* (Figure 3B). Exosomes were released in a much slower manner than CCL21a, and there were much less exosomes released from nanoGel than that from Gel at indicated time point (Figure 3B).

To investigate whether exosomes could be efficiently engulfed by cells in the hydrogel, exosomes were labeled with DiI before being loaded into the hydrogel. The distribution of DiI‐labeled exosomes uptaken by CT26.WT cells seeded in hydrogels was analyzed by fluorescence microscopy (Figure 3C). As shown in Figure 3D, there were accumulated DiI signals inside the DiO labeled CT26.WT cells, indicating efficient endocytosis of exosomes by CT26.WT cells in the hydrogel.

In view of the above data, we asked whether we could recruit the tumor cells to the hydrogel and then kill them there. To this end, Exo^GM‐CSF^ was additionally incubated with Ce6 before being loaded into the hydrogel. Briefly, 1 × 10^11^/mL Ce6 loaded exosomes and 400 ng mL^−1^ CCL21a or the controls were added into the nanoGel solution (Figure 3E). There was no significant difference in the release of CCL21a in CCL21a/Exo^GM‐CSF^@nanoGel and CCL21a@nanoGel, confirming that the release of CCL21a was not altered with the addition of exosomes in the hydrogel (Figure 3F). CT26.WT tumor‐bearing mice were injected with different hydrogels and treated with or without ultrasound irradiation, denoted as G1, G1′, G2, G2′, G3, and G4, respectively, on day 7 after tumor inoculation, followed by SDT three times on day 8. Cell apoptosis was determined by TUNEL on day 10 (Figure 3G). As expected, GFP^+^ tumor cells were efficiently recruited by CCL21a (Figure 3H,I). Compared with G1′ and G2′ groups, additional ultrasound irradiation in G1 and G2 groups, had no additional effects on cell death, suggesting that ultrasound alone without sonosensitizer had no killing effects. In contrast, in both G3 and G4 groups, there was abundant cell apoptosis, and many of them were CT26.WT‐GFP (Figure 3J). In addition, in the G4 group, there were abundant dendritic cells recruited, indicating the functional role of GM‐CSF mRNA in the exosomes. Notably, compared with the G2 group, there was an increase of DCs recruited to the gel in G3, suggesting that SDT alone could also recruit DCs (Figure S6, Supporting Information). Hematoxylin and eosin (H&E) staining revealed no significant changes in the histology of indicated tissues, further indicating that the hydrogels of different compositions possessed good biocompatibility (Figure S7, Supporting Information).

### 2.4 CCL21a/Exo^GM‐CSF+Ce6^@nanoGel/US Prevents Tumor Progression as a Potent Vaccine

**Figure 4:**
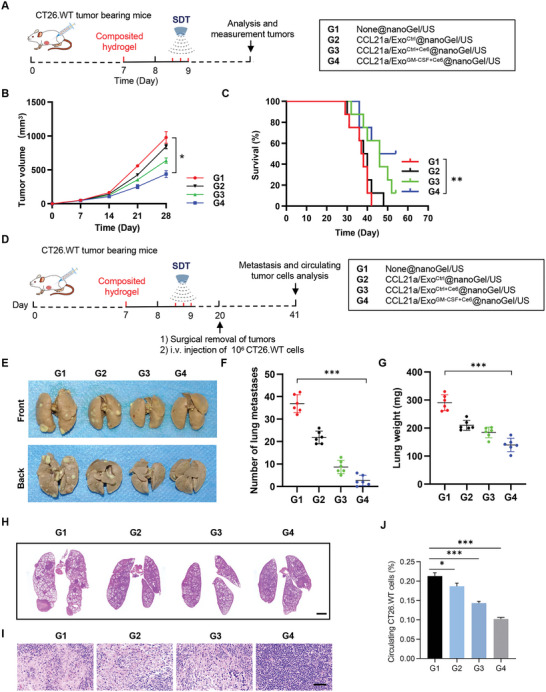
CCL21a/Exo^GM‐CSF+Ce6^@nanoGel/US prevents tumor progression in CT26.WT mouse model. A) Schematic illustration of the experimental procedure analyzing the tumor inhibition effects of the hydrogels. B) Tumor growth in mice treated with indicated hydrogels. Tumor size was monitored every 7 days. Data are expressed as mean ± S.E.M; *n* = 7–8 mice per group. *, *p* < 0.05 by two‐way ANOVA. C) Survival curves of CT26.WT tumor‐bearing mice treated with indicated hydrogels. *n* = 8 mice per group. **, *p* < 0.01 by log‐rank test. D) Schematic illustration of the experimental procedure analyzing metastasis prevention effects of the hydrogels. CT26.WT tumor‐bearing mice were randomly divided into four groups. On the 7th day of subcutaneous tumor inoculation in mice, hydrogels with different components were injected beside the tumor. Ultrasound irradiation was performed every 4 h for three times on day 8 after hydrogel injection. Tumors were surgically removed on the 20th day and the mice were challenged by i.v. injection of CT26.WT cells.The mice were sacrificed and lungs and tumor‐draining lymph nodes were harvested for further analysis on day 41. E) Representative images of lungs excised on day 41 in indicated groups. F) The number of lung metastatic nodules. Data are expressed as mean ± SD; *n* = 6. ***, *p* < 0.001 by one‐way ANOVA. G) Lung weights. Data are expressed as mean ± SD; *n* = 6. ***, *p* < 0.001 by one‐way ANOVA. H) Representative H&E staining of the lung sections from mice with indicated treatments. *n* = 6. Scale bar, 2000 µm. I) Representative H&E staining of the tumor‐draining lymph node sections from mice with indicated treatments. *n* = 6. Scale bar, 50 µm. J) Percentages of circulating CT26.WT‐GFP cells in peripheral blood from mice with indicated treatments. Data are expressed as mean ± S.E.M; *n* = 6. *, *p* < 0.05, ***, *p* < 0.001 by one‐way ANOVA.

We next asked whether CCL21a/Exo^GM‐CSF+Ce6^@nanoGel/US treatment could prevent/delay tumor progression in the CT26.WT syngeneic mouse model (**Figure** **4**A). As ultrasound irradiation has no obvious effects on cancer cell death and DC recruitment, mice were divided into four groups, all of which received ultrasound irradiation. In G4, the tumor volume was significantly smaller (Figure 4B), while the survival was significantly longer (Figure 4C). There were 4/8 of the mice survived more than 54 days in G4 and 1/8 of the mice survived in G3, whereas all mice in the other groups died within 29–48 days. Notably, the animals in G3 and G4 groups following the day 54‐time point were truly long‐term survivors, as no obvious tumor mass could be seen in these survived mice.

To further explore whether treatment in G4 could also prevent tumor metastasis, the primary tumor was surgically resected after the vaccine and then pulmonary metastasis was analyzed (Figure 4D). As shown in Figure 4E–G, there was less lung metastasis in G2, G3, and G4, with G4 having the most striking metastasis inhibition effects. Similarly, there was no significant TdLN metastasis observed in G4 (Figure 4H,I). Consistent with the findings in lung metastasis and lymph node metastasis, there were much fewer circulating cancer cells, as determined by the flow cytometry analysis of GFP‐positive cells in CT26.WT‐GFP syngeneic mouse model (Figure 4J and Figure S8, Supporting Information). These data suggested that CCL21a/Exo^GM‐CSF+Ce6^@nanoGel/US was an efficient vaccine with long‐term tumor prevention effects.

### 2.5 CCL21a/Exo^GM‐CSF+Ce6^@nanoGel/US Synergistically Promotes Dendritic Cell Maturation in CT26.WT Mouse Model

**Figure 5:**
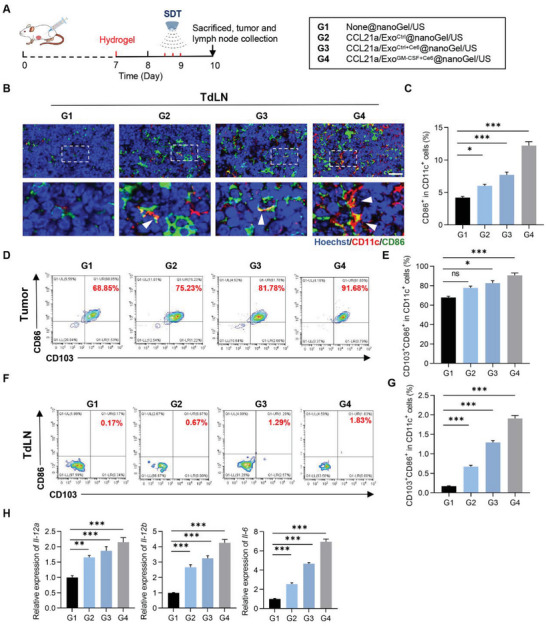
CCL21a/Exo^GM‐CSF+Ce6^@nanoGel/US promotes dendritic cell maturation in CT26.WT mouse model. A) Schematic representation of the experiment. CT26.WT colon tumor‐bearing mice were injected with indicated hydrogels beside the tumor on the 7th day. Ultrasound irradiation was performed every 4 h for three times on day 8. The mice were sacrificed and tumors and tumor‐draining lymph nodes were harvested for further analysis. B) Representative images of immunostaining of CD11c (red) and CD86 (green) in tumor‐draining lymph nodes from mice with indicated treatments. Scale bar, 30 µm. C) Statistical analysis of panel (B). Data are expressed as mean ± S.E.M, *n*=6. *, *p* < 0.05; ***, *p* < 0.001 by one‐way ANOVA. D) Representative flow cytometric analysis of mature cDC1 (CD11c^+^CD103^+^CD86^+^ cells) in tumors from mice with indicated treatments. E) Statistical analysis of panel (D). Data are expressed as mean ± S.E.M. *n* = 6. ns, no significance; *, *p* < 0.05; ***, *p* < 0.001 by one‐way ANOVA. F) Representative flow cytometric analysis of mature cDC1 (CD11c^+^CD103^+^CD86^+^ cells) in tumor‐draining lymph nodes from mice with indicated treatments. G) Statistical analysis of panel (F). Data are expressed as mean ± S.E.M. *n* = 6. ***, *p* < 0.001 by one‐way ANOVA. H) qPCR analysis of the expression of *Il‐12a, Il‐12b*, and *Il‐6* detected in tumor‐draining lymph nodes from mice with indicated treatments. Data are expressed as mean ± S.E.M. *n* = 3 biological replicates. **p < 0.01; ***p < 0.001 by one way ANOVA.

DCs play crucial roles in antigen presentation and T cell priming. It is well known that mature DCs highly express costimulatory molecules, such as CD80 and CD86, providing a second signal for the full activation of T cells.^[^ ^18^ ^]^ Hence, the effects of G4 treatment on DC maturation were analyzed by immunofluorescent microscope analysis and flow cytometry (**Figure** **5**A). As expected, there were more CD11c^+^ CD86^+^ cells in G4 than that in all other groups (Figure 5B,C). The frequency of matured DCs (CD11c^+^CD103^+^CD86^+^) in tumors from the G4 group was higher than that in other control groups (Figure 5D,E and Figure S9, Supporting Information). Similarly, CD11c^+^CD103^+^CD86^+^ DCs in TdLNs were also much higher in G4 (Figure 5F,G and Figure S10, Supporting Information). Consistently, G4 treatment greatly enhanced the production of the pro‐inflammatory factor IL‐6, IL‐12p35, and IL‐12p40 (Figure 5H), which may be attributed to the multi‐activation of DCs.^[^ ^19^ ^]^ These results suggested that G4 treatment‐induced tumor cell recruitment and ICD, DC recruitment, and maturation might synergistically amplify the innate immune response.

### 2.6 CCL21a/Exo^GM‐CSF+Ce6^@nanoGel/US Elicits T Cell Immune Response in CT26.WT Mouse Model

**Figure 6:**
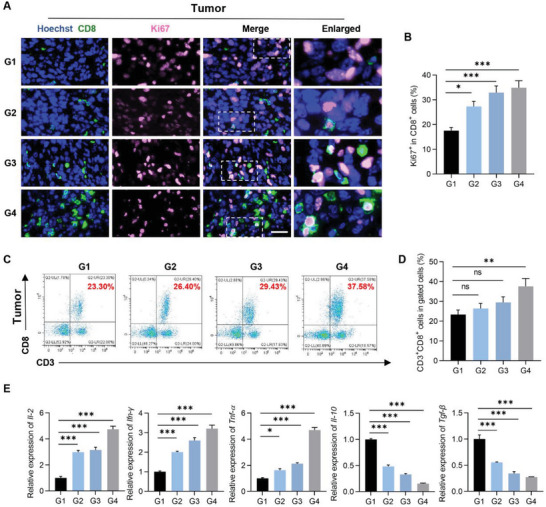
Activation of T cell antitumor immune response in CT26.WT mouse model. A) Representative images of immunostaining of CD8 (green) and Ki67 (pink) in inoculated tumors from mice with indicated treatments. Scale bar, 30 µm. B) Percentage of Ki67^+^ cells in CD8^+^ cells. Data are expressed as mean ± S.E.M. *n* = 6. *, *p* < 0.05; ***, *p* < 0.001 by one‐way ANOVA. C) Representative flow cytometric analysis of CTL (CD3^+^CD8^+^cells) in lymphocyte subpopulation from tumors from mice with indicated treatments. D) Statistical analysis of panel (C). Data are expressed as mean ± S.E.M. *n* = 6. ns, no significance; **, *p* < 0.01 by one‐way ANOVA. E) qPCR analysis of the expression of *Il‐2*, *Ifn‐γ*, *Tnf‐α*, *Il‐10*, and *Tgf‐β* detected in tumors from mice with indicated treatments. Data are representative of three different experiments and expressed as mean ± S.E.M. *, *p* < 0.05; ***, *p* < 0.001 by one way ANOVA.

We next explored whether treatment in G4 could enhance adaptive T cell immune responses. The frequency of CD3^+^CD4^+^ helper T cells and CD3^+^CD8^+^ cytotoxic lymphocytes (CTLs) in tumors and TdLNs were thus analyzed. Compared with the other three control groups, G4 treatment significantly promoted CD8^+^ cell proliferation, as seen by more Ki67^+^ in CD8^+^ cells in the tumors of G4 (**Figure** **6**A,B). Consistently, the proportion of CD3^+^CD8^+^ T cells in tumors was significantly greater in G4 than that in all other groups (Figure 6C,D). Nevertheless, there was no significant difference in the number of CD3^+^CD4^+^ T cells in the tumors among all four groups (Figure S11, Supporting Information). Notably, the ratios of CD8^+^ CTLs to CD4^+^ cells in tumors in G4 were the highest among all groups. Consistent with the enhanced CTL expansion, the proinflammatory cytokines interleukin‐2 (*Il‐2*), tumor necrosis factor (*Tnf*)‐*α*, and interferon (*Ifn*)‐*γ*, which promote antitumor immune response, were found significantly enhanced in tumor tissues after G4 treatment (Figure 6E). In contrast, expression of the immune‐suppressive *Il‐10*, and transforming growth factor‐b (*Tgf‐β*) were obviously decreased in tumors after G4 treatment (Figure 6E).

In accordance with the findings in tumors, the number of Ki67^+^ in CD3^+^ cells was highest in TdLNs of G4, in comparison with other groups (Figure S12A,B, Supporting Information). Moreover, there were decreased CD4^+^ T cells and enhanced CTL expansion observed in TdLNs in G4 (Figure S12C–F, Supporting Information). Taken together, these data indicated that CCL21a/Exo^GM‐CSF+Ce6^@nanoGel/US elicited efficient antitumor immunity in CT26.WT syngeneic mouse model.

### 2.7 CCL21a/Exo^GM‐CSF+Ce6^@nanoGel/US Elicits Strong Immune Response in 4T1 Syngeneic Breast Cancer Model

**Figure 7:**
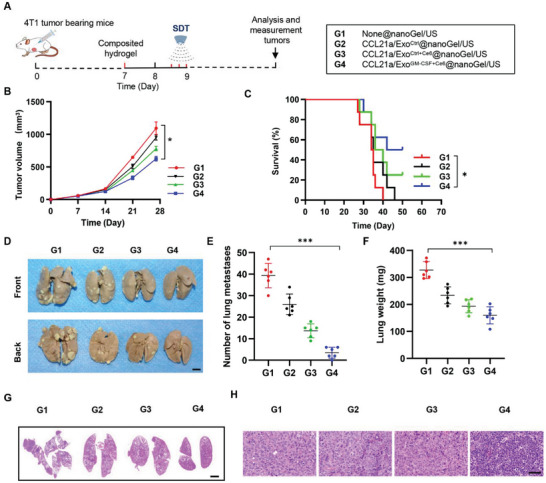
Antitumor effects of CCL21a/Exo^GM‐CSF+Ce6^@nanoGel/US in 4T1 breast cancer model. A) Schematic illustration of the experimental procedure evaluating the antitumor effects of G4 treatment in the 4T1 breast cancer model. B) Tumor growth in mice treated with indicated hydrogels. Tumor size was monitored every 7 days. Data are expressed as mean ± S.E.M; *n* = 7–8. *, *p* < 0.05 by two‐way ANOVA. C) Survival curves of 4T1 breast tumor‐bearing mice treated with indicated hydrogels. *n* = 8 mice per group. **, *p* < 0.01 by log‐rank test. D) Representative images of lungs excised on day 28. E) The number of lung metastatic nodules. Data are expressed as mean ± SD; *n* = 6. ***, *p* < 0.001 by one‐way ANOVA. F) Lung weights. Data are expressed as mean ± SD; *n* = 6. ***, *p* < 0.001 by one‐way ANOVA. G) Representative H&E staining of the lung sections from mice with indicated treatments. Mice were sacrificed on day 28. *n* = 6. Scale bar, 2000 µm. H) Representative H&E staining of the tumor‐draining lymph node sections from mice with indicated treatments. Mice were sacrificed on day 28. *n* = 6. Scale bar, 50 µm.

To further confirm the antitumor function of the G4 vaccine, we next explored the effects in the 4T1 syngeneic breast cancer mouse model (**Figure** **7**A). Consistent with the findings in CT26.WT model, the G4 vaccine significantly inhibited the tumor growth (Figure 7B) and prolonged the survival (Figure 7C) to a much higher extent than that in other groups. Notably, the mice in G3 and G4 following day 50 were truly long‐term survivors, as no obvious tumor mass could be seen in these survived mice. Accordingly, there was fewer lung and lymph node metastasis of 4T1 breast cancer in the group treated with G4 (Figure 7D–F). H&E staining further confirmed that G4 significantly suppressed lung and TdLN metastasis (Figure 7G,H).

We next analyzed DC maturation using flow cytometry and immunofluorescent microscopy (Figure S13A, Supporting Information). Compared with the control groups, G4 treatment also significantly enhanced the percentage of CD86^+^CD103^+^DCs in both tumors and TdLNs, suggesting that the G4 vaccine could promote DC maturation in the 4T1 mouse model (Figure S13B–E, Supporting Information). Accordingly, there was much higher expression of pro‐inflammatory cytokines, such as *Il‐6, Il‐12p35*, and *Il‐12p40*, in the G4 treatment group (Figure S13F, Supporting Information).

Consistent with the boosted DC maturation, G4 treatment significantly promoted CD8^+^ cell proliferation in the tumors, as seen by more Ki67^+^CD8^+^ cells in the tumors (Figure S14A,B, Supporting Information). The proportions of CD3^+^CD8^+^ T cells and CD3^+^CD4^+^ T cells in tumors were also significantly higher in the G4 vaccine group than those in all other groups (Figures S14C,D and S15, Supporting Information). Notably, the ratios of CD8^+^ CTLs to CD4^+^ cells in tumors were found to be the highest in the G4 group.

In accordance with the findings in tumors, the highest CD3^+^ T cell proliferation was found in TdLNs of the G4 vaccine group (Figure S16, Supporting Information). Enhanced CTL expansion was also observed in TdLNs (Figure S17A,B, Supporting Information). In contrast, the ratios of CD4^+^ cells in TdLNs were lower in the G4 group, which might be explained by the possible reduction of Treg cells (Figure S17C,D, Supporting Information). The proinflammatory cytokines *Il‐2*, *Tnf‐α*, and *Ifn‐γ* were found significantly enhanced in tumor tissues from mice with G4 vaccine treatment (Figure S18A, Supporting Information). In contrast, expression of the immune‐suppressive *Il‐10* and *Tgf‐β* were obviously decreased in tumors after the G4 vaccine (Figure S18B, Supporting Information).

Taken together, these data indicated that the engineered CCL21a/Exo^GM‐CSF+Ce6^@nanoGel together with ultrasound irradiation possessed the ability to elicit efficient antigen‐specific antitumor immunity in solid tumor models, demonstrating great potential for future clinical cancer vaccine development.

## 3 Discussion

In this study, we have developed a multifunctional hydrogel vaccine, namely CCL21a/Exo^GM‐CSF+Ce6^@nanoGel, for eliciting a powerful and sustained immune response. In the hydrogel system, direct encapsulation of CCL21a and exosome‐loaded GM‐CSF mRNA ensured CCL21a and GM‐CSF were released in distinct profiles. In the early stage, CCL21a was released and diverted TdLN metastatic tumor cells to the hydrogel. The trapped tumor cells then engulfed the Ce6‐containing exosomes and thus were eradicated by SDT, releasing the tumor antigens for DC presentation. In the later stage, the remnant CCL21a as the chemokine for DC homing and Exo^GM‐CSF+Ce6^ as the GM‐CSF and antigen source recruited DCs and elicited tumor‐specific immunotherapy. The proposed hydrogel system bypasses the insufficient DC/tumor antigen interaction in tumors and induces sustained immune response via topical induction of ICD and maturation of DCs synergistically, maximizing the vaccination efficacy. The strategy would open up an avenue for cancer immunotherapy.

Tumor‐antigen‐specific vaccines are being developed to treat some of the most devastating cancer types. Identification and characterization of the tumor‐associated antigens are time‐consuming and challenging, emerging as the bottleneck of tumor vaccine development.^[^ ^20^ ^]^ Among the vaccine strategies, cell‐based vaccines, including tumor whole‐cell vaccine or DC vaccine, have been intensively studied. For example, vaccines based on either patient‐derived tumor cells or allogenic tumor cells have been investigated for clinical use in patients.^[^ ^21^ ^]^ Recently, genetically modified tumor cells expressing costimulatory molecules have been developed. GVAX vaccines, genetically modified whole tumor cells expressing GM‐CSF, have shown promising results.^[^ ^22^ ^]^ Adoptive transfer of the DC vaccines is also increasingly explored. However, the outcomes are also challenged at least partially due to the insufficient survival of the transferred DC vaccine.^[^ ^23^ ^]^ Here, we developed a multifunctional hydrogel‐based vaccine comprising tumor cell recruitment cytokine, sonodynamic agent, DC recruitment cytokine, and exosome‐based tumor antigens, which could provoke potent and sustained immune responses. Compared with the adoptive transfer of activated DCs and subcutaneous administration of whole tumor cell vaccine, the proposed strategy might have superior advantages by orchestrating tumor cell recruitment, tumor cell eradication, DC recruitment, antigen expression, and DC maturation.

Hydrogels, which have high water content and mechanical tunability to resemble many biological tissues, are suitable for a variety of biomedical applications.^[^ ^24^ ^]^ Numerous hydrogel systems have been developed, especially for the controlled release of therapeutics, ranging from cytokine release to antigen/adjuvant delivery.^[^ ^25^ ^]^ In this study, CCL21a/Exo^GM‐CSF+Ce6^@nanoGel was designed to achieve multiple goals. Cell survival is a prerequisite for the following DC maturation and function. The GelMA based‐hydrogel is suitable for cell growth,^[^ ^10^, ^26^ ^]^ and is thus selected in this study to allow the recruited DCs to grow well in the hydrogel. It is well established that tumors promote immune cell apoptosis and paralyze antitumor immune response through the release of immune‐suppressive factors like NO, IL‐10, IL‐6, and arginase‐I, or shaping the DCs to a tumor‐suppressive phenotype.^[^ ^27^ ^]^ The hydrogel system bypasses the suppressive microenvironment limitation by establishing a novel microenvironment favoring tumor immunotherapy. The composited gel acts as a local stimulatory niche where infiltrating cells are exposed to high concentrations of adjuvant and antigens locally. Nanoclay, which is a kind of nanoparticle of layered mineral silicates, has been widely proposed as a biocompatible additive to refine the mechanical, thermal, and barrier properties of hydrogels.^[^ ^28^ ^]^ We here found that nanoclay incorporation facilitates the retention of exosomes, making the time gap between CCL21a‐mediated cancer cell recruitment and exosome‐based DC recruitment long enough.

In this study, the programmable release of CCL21a and GM‐CSF was designed to coordinately trap cancer cells and recruit DCs. CCL21a is a chemokine that promotes metastasis of many tumor types, including melanomas, breast, thyroid, colon, head, and neck cancers, via pairing with CCR7 expressed on tumor cells.^[^ ^13^, ^29^ ^]^ Release of CCL21a from the gel diverted the TdLN migrating tumor cells to hydrogel and thus blocked the TdLN metastasis theoretically. Moreover, tumor cells trapped in the hydrogel were eradicated by SDT, serving as antigen sources.

Later, the GM‐CSF release profile was achieved when the engineered exosomes encapsulated in the hydrogel were engulfed by the cells recruited. As we know, peptides/proteins are susceptible to degradation, and thus the life of the growth factor is rather short, limiting the immune activation duration.^[^ ^30^ ^]^ Exosomes are natural membrane systems that can protect the encapsulated mRNA from degradation.^[^ ^31^ ^]^ Extensive research on exosome‐based delivery platforms has confirmed that the system is good for mRNA delivery.^[^ ^32^ ^]^ Very recently, natural exosomes were found to be able to transfer tumor antigens, and exosomes could be also engineered as a therapeutic platform for designing DC vaccines.^[^ ^33^ ^]^ Our study has revealed that the tumor‐derived exosomes could be further engineered to encapsulate mRNA, serving as an antigen source and immune stimulator simultaneously. GM‐CSF mRNA in the exosomes embedded in the hydrogel would produce GM‐CSF protein when the exosomes are endocytosed by the recipient cells. Exo^GM‐CSF+Ce6^ in the nanogel thus could activate DCs via the released GM‐CSF from the exosome recipient cells. In turn, the maturation of DCs would migrate to the draining lymph nodes, where they prime the T cells. Together, the incorporated exosomes in the hydrogel system guarantee a durable capacity to recruit and elicit DC maturation.

ICD is a type of cancer cell death, which could release damage‐associated molecular patterns (DAMPs) and thus elicit the immune response against cancer. DAMPs include the cell surface exposure of calreticulin (CRT) and heat‐shock proteins (HSP70 and HSP90), extracellular release of adenosine triphosphate (ATP), high‐mobility group box‐1 (HMGB1), type I IFNs and members of the IL‐1 cytokine family.^[^ ^34^ ^]^ Since ultrasound can achieve deeper tissue penetration without damaging the surrounding healthy tissues, US‐triggered SDT emerges as an ideal method for cancer therapy. Upon local ultrasound, Ce6 produces toxic reactive oxygen species for ICD induction and thus activates antitumor immunity.^[^ ^35^ ^]^ Although the proposed hydrogel system preferentially recruits tumor cells at an earlier stage and DC cells in a delayed manner, some of the DCs would also have entered the material and been killed in the initial SDT. Different from systemic chemotherapy, which damages all the DCs in the whole body, SDT only kills a small population of the DCs in the hydrogel and most of the DCs in the circulation and lymph organs are protected. Theoretically, the death of these DCs in the hydrogel would not dampen the immune response significantly, as there would be more DCs recruited later especially when GM‐CSF was released. Future studies to specifically kill the cancer cells in the hydrogel will produce better therapeutic effects.

Surgical resection is the primary treatment for early‐stage and nonmetastatic solid tumors. However, tumor recurrence or metastasis post‐operation due to residual microtumors or circulating tumor cells is lethal to the patients.^[^ ^36^ ^]^ Routinely, chemotherapy or radiotherapy would be taken post‐surgery to eradicate the remnant cancer cells, though both therapies tend to decrease the life quality of patients. Alternatively, cancer immunotherapy has emerged as a powerful strategy to prevent tumor recurrence and metastasis.^[^ ^25^, ^32^, ^37^ ^]^ In this study, the proposed hydrogel‐based vaccine has potent efficacy to eradicate the circulating tumor cells and thus prevent cancer metastasis, suggesting the potential application of immunotherapy for the prevention of cancer recurrence and metastasis post‐surgery.

Future work to specifically recruit and kill the circulating tumor cells would be expected by optimizing the chemokines and the doses. Tailoring gel components favorable for DC activation would also further augment the therapeutic efficacy. Moreover, additional engineering of the exosomes to encapsulate more target mRNA with longer half‐life, incorporation of sonosensitizer with higher efficiency, and conjugation of the exosomes with hydrogel to prolong the retention without sacrificing the integrity, would be also needed.

## 4 Conclusion

In summary, we here have developed a programmable hydrogel vaccine, namely CCL21a/Exo^GM‐CSF+Ce6^@nanoGel, capable of eliciting a powerful and sustained immune response. Due to the programmable release of CCL21a and GM‐CSF, the hydrogel vaccine inhibits tumor growth and metastasis, via diverting cancer cell TdLNs metastasis to hydrogel, killing cancer cells trapped in the hydrogel to produce tumor antigen, and eliciting prolonged and powerful tumor‐specific immunotherapy coordinately. In summary, the engineered hydrogel system provides a holistic solution to maximize vaccine efficacy. The strategy we proposed herein might open an avenue for cancer immunotherapy, especially for the prevention of recurrence and metastasis post‐surgery.

## 5 Experimental Section

### Cell Culture

Mouse breast cancer cell 4T1 and colon cancer cell CT26.WT were purchased from Procell Life Science & Technology Co., Ltd. Dentric cell DC2.4 was purchased from FuHeng Cell Center. All cells were cultured in 1640 medium containing 10% FBS and 1% penicillin/streptomycin (P/S), with 5% CO_2_ at 37 °C.

### Plasmid Construction

The gene coding sequence of *CSF2* (also named GM‐CSF) was synthesized and cloned into pcDNA3.1(‐) by GenScript (Nanjing, China), with the resultant plasmid designated as pcDNA3.1(‐)‐GM‐CSF.

### Exosome Isolation and Additional Modification

Exosomes were isolated by ultracentrifugation. Briefly, exosome donor cells (4T1 or CT26) were transfected with control or pcDNA3.1(‐)‐GM‐CSF by Lipofectamine 2000 as instructed. 24 h later, cells were switched to a serum‐free medium and cultured for another 24 h. To remove dead cells and cell debris, the collected culture medium was centrifuged at 3000 g for 15 min. Then, the supernatant was centrifuged at 100 000 g for 2 h to obtain the exosomes, with the derived exosomes named Exo^Ctrl^ or Exo^GM‐CSF^, respectively. The isolated exosomes were resuspended with PBS and stored at −80 °C till use.

For the preparation of Exo^Ctrl+Ce6^/Exo^GM‐CSF+Ce6^, 10 µL of Ce6 (15 µg µL^−1^) (Cayman Chemical, Ann Arbor, Michigan) was incubated with 300 µg purified Exo^Ctrl^ or Exo^GM‐CSF^ for 2 h at 37 °C. Hydrophobic Ce6 could be incorporated into the lipid membrane of exosomes and the free Ce6 was removed by additional exosome isolation with centrifugation at 12 000 g for 30 min.

### Exosome Characterization

Exosomes particle size and concentration were measured by NanoSight (Malvern, UK). Morphology was visualized using an electron microscope (JEM‐2000EX TEM; JEOL, Tokyo, Japan). Briefly, the exosomes were added onto the grid and stained with methyl cellulose‐uranyl acetate (100 µL of 4% uranyl acetate and 900 µL of 2% methyl cellulose), followed by observation under the transmission electron microscope when air dried.

### Western Blot

Tissue/cell/exosome samples were prepared by RIPA Lysis Buffer (Beyotime Biotechnology, China), and the protein concentration was measured by BCA Protein Assay Kit (Thermo Scientific, Somerset, NJ). Proteins were then separated by SDS‐PAGE (10%) and transferred to nitrocellulose membranes. After blocking with 5% fat‐free milk, the membranes were subsequently incubated with first antibodies at 4 °C including anti‐GM‐CSF (17762‐1‐AP, ProteinTech), anti‐GM130 (11308‐1‐AP, ProteinTech), anti‐TSG101 (ab83, Abcam), anti‐CD9 (ab92726, Abcam), and anti‐GAPDH (D110016‐0100, BBI Life Sciences). The membranes were washed three times in TBST to remove free peptides before incubation with the secondary antibodies (anti‐mouse [7076, CST] or anti‐rabbit [7074, CST]) in tris‐buffered saline at room temperature for 1 h. The protein bands were visualized with chemiluminescence (GE Healthcare, Chalfont St. Giles, UK). All the experiments were performed at least three times.

### Real‐Time PCR

Total RNA was extracted using TRIzol reagent (Invitrogen, USA). Reverse transcription with 2 µg RNA was conducted with the PrimeScript First‐Strand cDNA Synthesis Kit (Takara, China). Real‐time PCR was performed using FastStart Essential DNA Green Master. The target mRNA expression was normalized to *Gapdh*. The relative gene expression was calculated with the 2^‐ΔΔCt^ method. The primer sequences for *Gm‐csf*, *Il‐2*, *Il‐10*, *Tgf‐b*, *Il‐12a*, *Il12b*, *Ifn‐γ*, *Tnf‐α*, *Il‐6*, and *Gapdh* are listed in Table S1, Supporting Information.

### Hydrogel Preparation and Characterization

Lithium phenyl‐2,4,6‐ trimethylbenzoylphosphinate (LAP) solution (0.25% w/v) was used for hydrogel crosslinking. Briefly, 0.05 g LAP was dissolved with 20 mL PBS at 45 °C. GelMA (EFL‐GM‐60, China) was dissolved to 10% (w/v) with LAP solution at 65 °C, followed by addition of 0.04 g mL^−1^ nanoclay (hydrophilic bentonite, Sigma‐Aldrich 682 659, CAS Number:1302‐78‐9) for construction of nanoGel. The mixture was incubated at 37 °C for 2 h. The composited hydrogel was then crosslinked for 30 s using blue light. For reticular porous structure analysis, the hydrogels with different compositions were lyophilized and cut into pieces in a longitudinal direction. The specimens were sputter‐coated with gold in a vacuum for SEM examination (Quattro S, Thermo Fisher Scientific, US).

### Rheological Properties and Degradation Behavior Assay

The rheological properties of GelMA and GelMA/nanoclay were measured by a rheometer (Discovery HR‐2, TA). To evaluate the degradation behaviors of hydrogels, the samples (5 mm height; 10 mm diameter; *n* = 3) were soaked in PBS at 37 °C. The weight of hydrogels was measured at given time points (days 1 to 14) after removing the surface supernatant. The following formula was used to calculate the degradation ratios of hydrogels: Degradation ratio = *W*_0_−*W*_t_/*W*_0_ × 100%, where *W*_0_ is the initial weight of the sample, and *W*_t_ is the weight of the sample at a specific time point.

### Release of CCL21a and Exosomes from the Hydrogel

To evaluate the release profile of CCL21a and exosomes from the hydrogels composited of GelMA or GelMA/nanoclay, about 300 µg exosomes and/or 80 ng CCL21a was dissolved in 200 µL GelMA or GelMA/nanoclay solution, followed by mixing thoroughly and gelation as described above. The resultant hydrogels were then incubated in PBS at 37 °C. The supernatant was collected at indicated time points, and meanwhile, the same amount of fresh PBS was added. The released CCL21a was examined by ELISA kit (EMCCL21A, Invitrogen) as instructed. The released exosomes were examined by qPCR analysis of the encapsulated Gapdh. The cumulative release was then calculated.

### ELISA Assay of GM‐CSF

For cytokine production analysis, 2 × 10^6^ recipient cells were treated with 40 µg Exo^None^, Exo^Ctrl^, or Exo^GM‐CSF^, and 24 h later, cell‐free supernatants were collected for analysis of the level of GM‐CSF using an ELISA kit (BMS612, Invitrogen) as instructed.

### Exosome Uptake by CT26 Cells in Hydrogel

Exosomes (1 mg mL^−1^) were labeled with DiI dye as described before.^[^ ^38^ ^]^ Then, 300 µg labeled exosomes were dissolved in 200 µL hydrogel with different composites. DiO labeled CT26.WT cells were co‐cultured with exosome‐embed hydrogel for 24 h. Then the hydrogels were fixed in 4% paraformaldehyde (PFA) for 20 min, followed by sectioning. Cell nuclei were counterstained with Hoechst and the phagocytosis of exosomes was observed under Nikon A1 Spectral Confocal Microscope (Nikon, Japan).

### Cellular Toxicity of Hydrogel

Calcein‐AM and propidium iodide (PI) (C2015M, Beyotime) were applied for live and dead cell staining, respectively. CT26.WT‐GFP or DC2.4 cells were cultured in hydrogels with different components in a confocal dish. The live/dead solution was freshly prepared and added to each confocal dish and incubated for 30 min at 37 °C at the indicated time of culture. The fluorescence signal was examined under a Nikon A1 Spectral Confocal Microscope (Nikon, Japan). The hydrogels were degraded with GelMA lysis solution (EFL‐GM‐LS‐001, EFL, China) at 37 °C for 2 h. The number of cells in the gel was then counted with Celldrop FL after isolation (DENOVIX, US).

### Animal Housing, Tumor Inoculation, and Hydrogel‐Based Vaccine

6‐ to 8‐week‐old Balb/c mice were purchased from the Lab Animal Center of the Fourth Military Medical University. The mice were housed in 3–5 per cage under specific pathogen‐free conditions in a 12‐h light/dark cycle with food and water ad libitum. The experiments were approved by the Institutional Animal Experiment Administration Committee of the Fourth Military Medical University (20210100635‐2).

The 4T1 syngeneic mouse model was established by implanting 1 × 10^5^ 4T1 cancer cells in 100 µL of PBS into the right second mammary fat pads of the Balb/c mice. The colon carcinoma mouse model was established by subcutaneously implanting 1 × 10^6^ CT26.WT or CT26.WT‐GFP cancer cells in the right flank of the Balb/c mice. Tumor growth was monitored by palpation every 7 days. Tumor size was calculated as *V* = *a* × *b* ^2^/2, where *a* indicates the longer diameter and *b* indicates the shorter diameter.

For hydrogel‐based vaccination, 200 µL hydrogel solution with different components was injected beside the tumor on the 7th day of subcutaneous tumor inoculation, followed by gelation. Briefly, tumor bearing mice were subcutaneously injected with None@nanoGel group (G1′), None@nanoGel/US group (G1), CCL21a/Exo^Ctrl^@nanoGel group (G2′), CCL21a/Exo^Ctrl^@nanoGel/US group (G2), CCL21a/Exo^Ctrl+Ce6^@nanoGel/US group (G3), and CCL21a/Exo^GM‐CSF+Ce6^@nanoGel/US group (G4). For the 4T1 syngeneic mouse model, exosomes from 4T1 cells were used, and exosomes from CT26 cells were used for CT26 syngeneic mouse model. Gelation was induced by blue light irradiation for 3 min (Figure S3, Supporting Information). For SDT, ultrasound irradiation (frequency of 1 MHz, power density of 2 W cm^−2^, 20% duty cycle for 5 min) was performed in the hydrogel injected region every 4 h for three times on day 8. The mice were sacrificed and tumors and TdLNs were harvested for histology and immune response analysis.

### Immunofluorescence, H&E, and TUNEL

TdLNs, tumors, and hydrogels were collected and embedded in an optimal cutting temperature (OCT) compound for frozen sections using a cryostat. Next, 8‐µm slides were cut and fixed with 4% PFA. For immunofluorescence analysis, slides were blocked with 5% bovine serum albumin and then incubated with the primary antibodies (anti‐CD3, 1:3000, Servicebio, China, GB13014; anti‐CD4, 1:3000, Servicebio, China, GB13064‐2; anti‐CD8, 1:3000, Servicebio, China, GB13429; anti‐CD11c, 1:400, Servicebio, China, GB11059; anti‐CD86, 1:4000, Servicebio, China, GB13586; anti‐Ki67, 1:1000, Servicebio, China, GB111141) overnight at 4 °C. After three washes with PBS, the samples were incubated with the secondary antibody (Cy3‐goat anti‐rabbit, 1:300, Servicebio, China, GB21303; 488‐goat anti‐rabbit, 1:300, Servicebio, China, GB25303) for 2 h at room temperature. Cell nuclei of the tissues were counterstained with Hoechst and the slices were observed with a Nikon A1 Spectral Confocal Microscope (Nikon, Japan). Similar procedures were performed for cell death analysis using the TUNEL kit (C1090, Beyotime) as instructed by the protocol. For quantification of cells of interest in each group, 15 slices of the tissues from three mice in each group were stained. The images of the whole slides were captured and cells of interest were counted from randomly chosen 20 fields. Cell counting was performed in a design‐blind manner.

For H&E staining, TdLNs, tumors, hearts, spleens, lungs, livers, kidneys, and hydrogels were collected and fixed with 4% PFA. The samples were embedded in paraffin and cut into 5 µm slides. The sections were then stained with H&E as instructed.

### Flow Cytometry

TdLNs and tumors from mice with different treatments were collected for flow cytometry analysis. Briefly, single‐cell suspensions were prepared by digestion with collagenase type IV (2.5 mg mL^−1^, Gibco, USA) in Hank's Balanced Salt Mixture containing calcium and magnesium (Solarbio, China) for 1.5 h at 37 °C. Next, the cells were then diluted to a concentration of 1 × 10^7^ cells/mL in a Cell Staining Buffer (BioLegend, USA). Cell samples were then stained with anti‐CD3‐PE, anti‐CD4‐APC‐Cy7, anti‐CD8‐APC, anti‐CD11c‐APC, anti‐CD86‐PerCP, and anti‐103‐PE for 1 h in the dark (BioLegend, USA). Then, the cells were washed twice with Cell Staining Buffer, and analyzed with Beckman CytoFLEX. Flow cytometry data were analyzed using CytExpert software. Percentages of CTLs (CD3^+^CD4^‐^CD8^+^), helper T cells (CD3^+^CD4^+^CD8^‐^), and mature dendritic cells (CD11c^+^CD103^+^CD86^+^) were calculated.

### Statistical Analysis

All the data are presented as mean ± S.E.M or mean ± SD as indicated. The unpaired Student's *t*‐test was used for the statistical analysis of the two groups. One‐way analysis of variance (ANOVA) with Tukey's post hoc test for analysis among multiple groups. The statistical differences of double factors were analyzed by two‐way ANOVA with Tukey's post hoc test. Statistical analysis was done by GraphPad Prism 7.0 and significant differences are considered at *p* < 0.05.

## Conflict of Interest

The authors declare no conflict of interest.

## Author Contributions

P.J., W.S., and S.Z. contributed equally to this work. G.Y., G.J., and Q.L. designed the research. P.J., W.S., S.Z., Y.X., C.W., and M.W. performed the research. All authors analyzed and interpreted the data. G.Y. and P.J. wrote the paper.

## Supporting information

Supporting Information

## Data Availability

The data that support the findings of this study are available from the corresponding author upon reasonable request.

## References

[1] a) A. D. Waldman , J. M. Fritz , M. J. Lenardo , Nat. Rev. Immunol. 2020, 20, 651; 32433532 10.1038/s41577-020-0306-5 PMC7238960

[2] a) S. Yu , C. Wang , J. Yu , J. Wang , Y. Lu , Y. Zhang , X. Zhang , Q. Hu , W. Sun , C. He , X. Chen , Z. Gu , Adv. Mater. 2018, 30, 1801527; 10.1002/adma.201801527 29786888

[3] a) M. Saxena , S. H. van der Burg , C. J. M. Melief , N. Bhardwaj , Nat. Rev. Cancer 2021, 21, 360; 33907315 10.1038/s41568-021-00346-0

[4] a) D. F. Quail , J. A. Joyce , Nat. Med. 2013, 19, 1423; 24202395 10.1038/nm.3394 PMC3954707

[5] S. K. Wculek , F. J. Cueto , A. M. Mujal , I. Melero , M. F. Krummel , D. Sancho , Nat. Rev. Immunol. 2020, 20, 7. 31467405 10.1038/s41577-019-0210-z

[6] J. M. T. Janco , P. Lamichhane , L. Karyampudi , K. L. Knutson , J. Immunol. 2015, 194, 2985. 25795789 10.4049/jimmunol.1403134 PMC4369768

[7] a) F. Lang , B. Schrörs , M. Löwer , Ö. Türeci , U. Sahin , Nat. Rev. Drug Discovery 2022, 21, 261; 35105974 10.1038/s41573-021-00387-y PMC7612664

[8] a) K. Palucka , J. Banchereau , Nat. Rev. Cancer 2012, 12, 265; 22437871 10.1038/nrc3258 PMC3433802

[9] a) S. Correa , A. K. Grosskopf , H. L. Hernandez , D. Chan , A. C. Yu , L. M. Stapleton , E. A. Appel , Chem. Rev. 2021, 121, 11385; 33938724 10.1021/acs.chemrev.0c01177 PMC8461619

[10] K. Yue , G. Trujillo‐de Santiago , M. M. Alvarez , A. Tamayol , N. Annabi , A. Khademhosseini , Biomaterials 2015, 73, 254. 26414409 10.1016/j.biomaterials.2015.08.045 PMC4610009

[11] a) E. I. Buzas , Nat. Rev. Immunol. 2022, 23, 236; 35927511 10.1038/s41577-022-00763-8 PMC9361922

[12] a) C. Théry , L. Zitvogel , S. Amigorena , Nat. Rev. Immunol. 2002, 2, 569; 12154376 10.1038/nri855

[13] a) A. Müller , B. Homey , H. Soto , N. Ge , D. Catron , M. E. Buchanan , T. McClanahan , E. Murphy , W. Yuan , S. N. Wagner , J. L. Barrera , A. Mohar , E. Verástegui , A. Zlotnik , Nature 2001, 410, 50; 11242036 10.1038/35065016

[14] J. M. Lee , M. H. Lee , E. Garon , J. W. Goldman , R. Salehi‐Rad , F. E. Baratelli , D. Schaue , G. Wang , F. Rosen , J. Yanagawa , T. C. Walser , Y. Lin , S. J. Park , S. Adams , F. M. Marincola , P. C. Tumeh , F. Abtin , R. Suh , K. L. Reckamp , G. Lee , W. D. Wallace , S. Lee , G. Zeng , D. A. Elashoff , S. Sharma , S. M. Dubinett , Clin. Cancer Res. 2017, 23, 4556. 28468947 10.1158/1078-0432.CCR-16-2821 PMC5599263

[15] W. Zhao , G. Zhao , B. Wang , Cell Mol. Immunol. 2018, 15, 187. 29057973 10.1038/cmi.2017.105 PMC5811680

[16] H. M. Zhu , Y. He , S. S. Huang , J. J. Tian , L. S. Wang , J. D. Hao , B. Xie , J. J. Ling , Pharm. Dev. Technol. 2020, 25, 1249. 32811263 10.1080/10837450.2020.1810274

[17] I. Li , B. Y. Nabet , Mol. Cancer 2019, 18, 32. 30823926 10.1186/s12943-019-0975-5 PMC6397467

[18] a) K. Liu , G. D. Victora , T. A. Schwickert , P. Guermonprez , M. M. Meredith , K. Yao , F. F. Chu , G. J. Randolph , A. Y. Rudensky , M. Nussenzweig , Science 2009, 324, 392; 19286519 10.1126/science.1170540 PMC2803315

[19] P. Nair‐Gupta , A. Baccarini , N. Tung , F. Seyffer , O. Florey , Y. Huang , M. Banerjee , M. Overholtzer , P. A. Roche , R. Tampé , B. D. Brown , D. Amsen , S. W. Whiteheart , J. M. Blander , Cell 2014, 158, 506. 25083866 10.1016/j.cell.2014.04.054 PMC4212008

[20] a) S. Bobisse , R. Genolet , A. Roberti , J. L. Tanyi , J. Racle , B. J. Stevenson , C. Iseli , A. Michel , M. A. Le Bitoux , P. Guillaume , J. Schmidt , V. Bianchi , D. Dangaj , C. Fenwick , L. Derré , I. Xenarios , O. Michielin , P. Romero , D. S. Monos , V. Zoete , D. Gfeller , L. E. Kandalaft , G. Coukos , A. Harari , Nat. Commun. 2018, 9, 1092; 29545564 10.1038/s41467-018-03301-0 PMC5854609

[21] B. P. Keenan , E. M. Jaffee , Semin. Oncol. 2012, 39, 276. 22595050 10.1053/j.seminoncol.2012.02.007 PMC3356993

[22] a) J. Nemunaitis , Expert Rev. Vaccines 2005, 4, 259; 16026242 10.1586/14760584.4.3.259

[23] a) K. Palucka , J. Banchereau , Immunity 2013, 39, 38; 23890062 10.1016/j.immuni.2013.07.004 PMC3788678

[24] a) J. Li , D. J. Mooney , Nat. Rev. Mater. 2016, 1, 16071; 29657852 10.1038/natrevmats.2016.71 PMC5898614

[25] a) Y. Wu , P. K. Norberg , E. A. Reap , K. L. Congdon , C. N. Fries , S. H. Kelly , J. H. Sampson , V. P. Conticello , J. H. Collier , ACS Biomater. Sci. Eng. 2017, 3, 3128; 30740520 10.1021/acsbiomaterials.7b00561 PMC6364304

[26] a) P. Occhetta , R. Visone , L. Russo , L. Cipolla , M. Moretti , M. Rasponi , J. Biomed. Mater. Res., Part A 2015, 103, 2109; 10.1002/jbm.a.35346 25294368

[27] a) B. R. Gastman , D. E. Johnson , T. L. Whiteside , H. Rabinowich , Blood 2000, 95, 2015; 10706869

[28] a) B. Liu , J. Li , X. Lei , S. Miao , S. Zhang , P. Cheng , Y. Song , H. Wu , Y. Gao , L. Bi , G. Pei , RSC Adv. 2020, 10, 25652; 35518607 10.1039/d0ra03040f PMC9055310

[29] a) R. Förster , A. C. Davalos‐Misslitz , A. Rot , Nat. Rev. Immunol. 2008, 8, 362; 18379575 10.1038/nri2297

[30] a) X. Huang , N. Kong , X. Zhang , Y. Cao , R. Langer , W. Tao , Nat. Med. 2022, 28, 2273; 36357682 10.1038/s41591-022-02061-1

[31] a) R. Kalluri , V. S. LeBleu , Science 2020, 367, eaau6977; 32029601 10.1126/science.aau6977 PMC7717626

[32] a) D. Ha , N. Yang , V. Nadithe , Acta Pharm. Sin. B 2016, 6, 287; 27471669 10.1016/j.apsb.2016.02.001 PMC4951582

[33] R. Hosseini , L. Asef‐Kabiri , H. Yousefi , H. Sarvnaz , M. Salehi , M. E. Akbari , N. Eskandari , Mol. Cancer 2021, 20, 83. 34078376 10.1186/s12943-021-01376-w PMC8170799

[34] A. Ahmed , S. W. G. Tait , Mol. Oncol. 2020, 14, 2994. 33179413 10.1002/1878-0261.12851 PMC7718954

[35] a) H. Zhao , B. Zhao , L. Li , K. Ding , H. Xiao , C. Zheng , L. Sun , Z. Zhang , L. Wang , Adv. Healthcare Mater. 2020, 9, 1901335; 10.1002/adhm.201901335 31762228

[36] a) S. Paik , S. Shak , G. Tang , C. Kim , J. Baker , M. Cronin , F. L. Baehner , M. G. Walker , D. Watson , T. Park , W. Hiller , E. R. Fisher , D. L. Wickerham , J. Bryant , N. Wolmark , N. Engl. J. Med. 2004, 351, 2817; 15591335 10.1056/NEJMoa041588

[37] a) C. Wang , W. Sun , G. Wright , A. Z. Wang , Z. Gu , Adv. Mater. 2016, 28, 8912; 27558441 10.1002/adma.201506312 PMC5283805

[38] P. Ji , Z. Yang , H. Li , M. Wei , G. Yang , H. Xing , Q. Li , Mol. Ther.–Nucleic Acids 2021, 26, 987. 34760340 10.1016/j.omtn.2021.10.009 PMC8560825

