# Supplementary material for: Modular Hydrogel Vaccine for Programmable and Coordinate Elicitation of Cancer Immunotherapy
Source: Adv Sci (Weinh). 2023 May 24;10(22):2301789. doi: 10.1002/advs.202301789 (PMC10401092; doi:10.1002/advs.202301789)
Supplement: Supplementary file 1 — Supporting Information [file ADVS-10-2301789-s001.pdf]

ADVANCED SCIENCE
Open Access

Supporting Information

for *Adv. Sci.*, DOI 10.1002/advs.202301789

Modular Hydrogel Vaccine for Programmable and Coordinate Elicitation of Cancer Immunotherapy

Panpan Ji, Wenqi Sun, Siyan Zhang, Yuqi Xing, Chen Wang, Mengying Wei, Qiuyun Li, Gang Ji* and Guodong Yang**

ADVANCED SCIENCE
Open Access

Supporting Information

for *Adv. Sci.*, DOI 10.1002/advs.202301789

Modular Hydrogel Vaccine for Programmable and Coordinate Elicitation of Cancer Immunotherapy

Panpan Ji, Wenqi Sun, Siyan Zhang, Yuqi Xing, Chen Wang, Mengying Wei, Qiuyun Li, Gang Ji* and Guodong Yang**

Supporting Information

Modular hydrogel vaccine for programmable and coordinate elicitation of cancer immunotherapy

Panpan Ji, Wenqi Sun, Siyan Zhang, Yuqi Xing, Chen Wang, Mengying Wei, Qiuyun Li*, Gang Ji*, Guodong Yang*

P.Ji, G.Ji
Department of Digestive Surgery, Xijing Hospital, Fourth Military Medical University, Shaanxi, 710032, China.
E-mail: jigang@fmmu.edu.cn

W.Sun, S.Zhang, C.Wang
Department of Ultrasound Diagnostics, Tangdu Hospital, Fourth Military Medical University, Shaanxi, 710038, China.

Y.Xing, M.Wei, G.Yang
The State Laboratory of Cancer Biology, Department of Biochemistry and Molecular Biology, Fourth Military Medical University, Shaanxi, 710032, China.
E-mail: yanggd@fmmu.edu.cn

Q.Li
Department of Breast Surgery, The Affiliated Tumor Hospital of Guangxi Medical University, Nanning, Guangxi, 530021, China.
E-mail: qyli1020@sina.com

Keywords: Cancer immunotherapy; Vaccine; Hydrogel; Programmed release; Antigen presentation; Exosomes

Table S1. Primers used in this study.

**qPCR Primers**		
Gm-csf	Forward	5’-GGCCTTGGAAGCATGTAGAGG-3'
	Reverse	5’-GGAGAACTCGTTAGAGACGACTT-3'
Il-2	Forward	5'-TGAGCAGGATGGAGAATTACAGG-3'
	Reverse	5'-GTCCAAGTTCATCTTCTAGGCAC-3'
Ifn-γ	Forward	5'-ATGAACGCTACACACTGCATC-3'
	Reverse	5'-CCATCCTTTTGCCAGTTCCTC-3'
Tnf-α	Forward	5’-CCCTCACACTCAGATCATCTTCT-3’
	Reverse	5’-GCTACGACGTGGGCTACAG-3’
Il-10	Forward	5’-GCTCTTACTGACTGGCATGAG-3’
	Reverse	5’-CGCAGCTCTAGGAGCATGTG-3’
Tgf-β	Forward	5’-CTCCCGTGGCTTCTAGTGC-3’
	Reverse	5’-GCCTTAGTTTGGACAGGATCTG -3’
Il-6	Forward	5’-TAGTCCTTCCTACCCCAATTTCC-3’
	Reverse	5’-TTGGTCCTTAGCCACTCCTTC-3’
Il-12a	Forward	5’-CTGTGCCTTGGTAGCATCTATG-3’
	Reverse	5’-GCAGAGTCTCGCCATTATGATTC-3’
Il-12b	Forward	5’-TGGTTTGCCATCGTTTTGCTG-3’
	Reverse	5’-ACAGGTGAGGTTCACTGTTTCT-3’
Gapdh	Forward	5’-AGGTCGGTGTGAACGGATTTG-3’
	Reverse	5’-TGTAGACCATGTAGTTGAGGTCA-3’

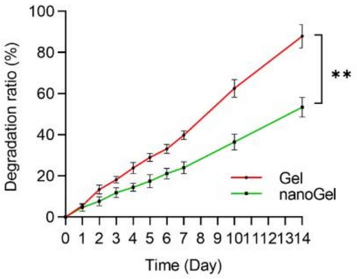

Figure S1. Degradation ratio of Gel or nanoGel. Data are expressed as mean ±S.E.M of three independent experiments. **, p< 0.01 by two-way ANOVA.

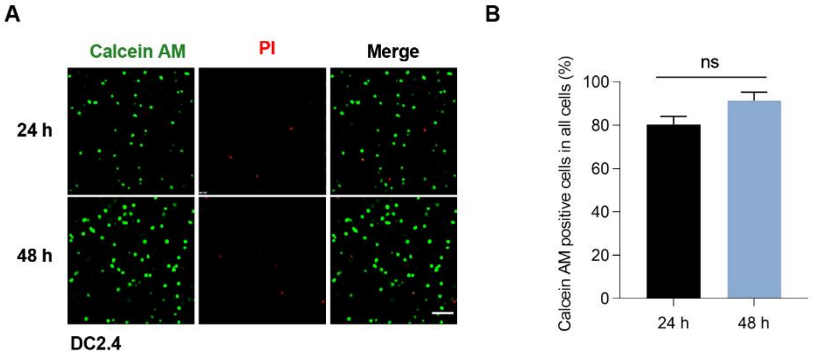

Figure S2. Viability of DC2.4 in nanoGel. (A) DC2.4 were cultured in nanoGel and cell viability was measured by Calcein-AM/PI staining at the indicated time. Calcein-AM, green; PI, red. Scale bar, 100 μm. (B) The hydrogels were treated with gel lysis solution at 37 °C and the Calcein AM^+ DC2.4 cells were then counted. Data are expressed as mean ±S.E.M of three independent experiments. ns, no significance by t-test.

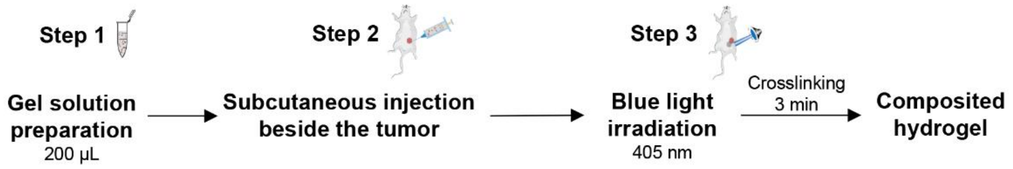

Figure S3. Schematic showing the experimental procedure. GelMA solution with different components is prepared and subcutaneously injected beside the tumor. Gelation is achieved by light irradiation in the injection site.

GGATCC**GCCACC**ATGTGGCTGCAGAATTTACTTTTCCTGGGCATTGTGGTCTACAGCCTCTCAGCACCCACCCGCTCACC
CATCACTGTCACCCGGCCTTGGAAGCATGTAGAGGCCATCAAAGAAGCCCTGAACCTCCTGGATGACATGCCTGTCACGT
TGAATGAAGAGGTAGAAGTCGTCTCTAACGAGTTCTCCTTCAAGAAGCTAACATGTGTGCAGACCCGCCTGAAGATATTCG
AGCAGGGTCTACGGGGCAATTTCACCAAACTCAAGGGCGCCTTGAACATGACAGCCAGCTACTACCAGACATACTGCCCC
CCAACTCCGGAAACGGACTGTGAAACACAAGTTACCACCTATGCGGATTTCATAGACAGCCTTAAAACCTTTCTGACTGATA
TCCCCTTTGAATGCAAAAAACCAGGCCAAAAATGA**AAGCTT**

Figure S4. Plasmid construction information. The Kozak sequence is shown in blue shadow and the restriction enzyme sites are shown in purple.

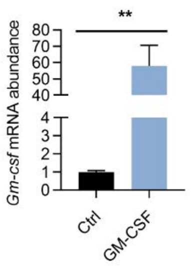

Figure S5. *Gm-csf* mRNA abundance in recipient cells treated as indicated. *Gapdh* served as an internal control. Data are expressed as mean ± S.E.M of three independent experiments. **, p< 0.01 by t-test.

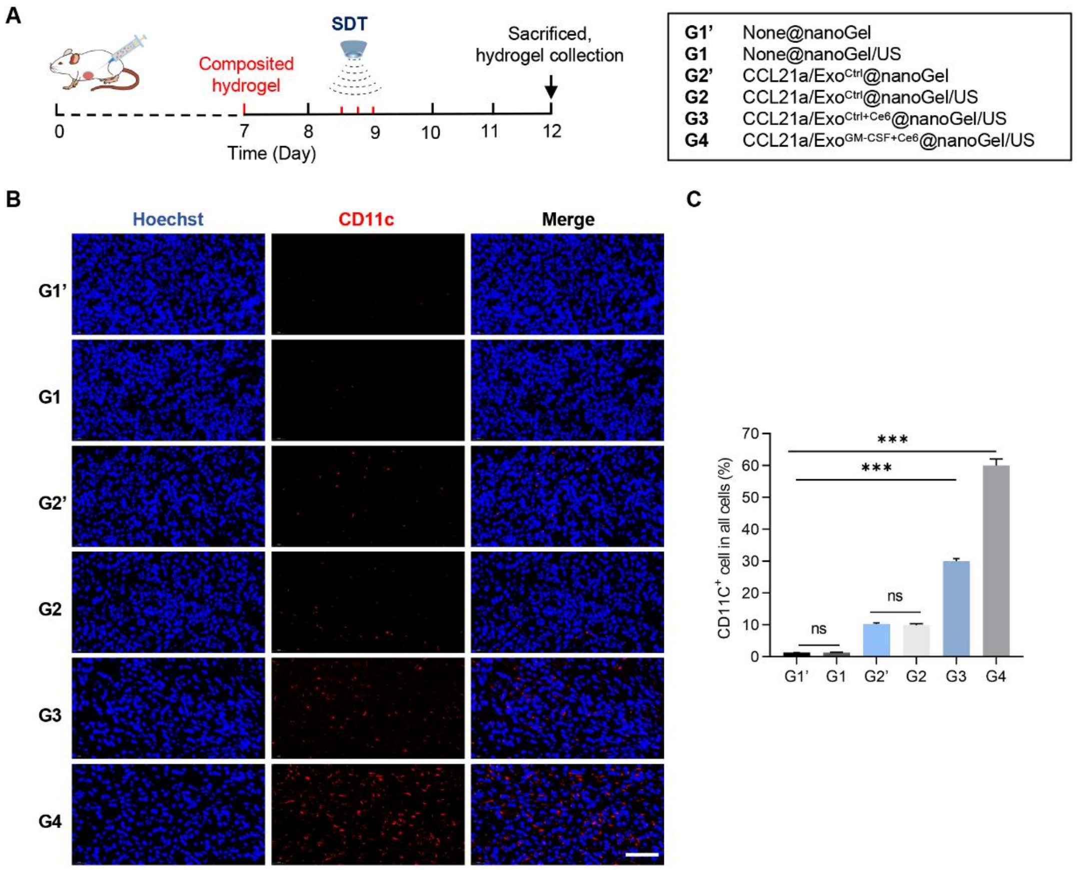

Figure S6. Chemotaxis of dendritic cells towards hydrogels. (A) Schematic illustration of the experimental procedure analyzing the chemotactic effects of hydrogel on DC cells. CT26.WT-GFP tumor bearing mice were randomly divided into six groups, None@nanoGel (G1'), None@nanoGel group/US (G1), CCL21a/ Exo^{Ctrl}@nanoGel (G2'), CCL21a/Exo^{Ctrl}@nanoGel/US group (G2), CCL21a/ $Exo^{Ctrl+Ce6}$@nanoGel/US group (G3) and CCL21a/$Exo^{GM\text{-}CSF+Ce6}$@nanoGel/US group (G4). On the 7th day of subcutaneous tumor inoculation, indicated hydrogels were injected beside the tumor. Ultrasound irradiation was performed every 4 hours for three times on day 8. The mice were sacrificed and hydrogels were harvested for further analysis. (B) Representative fluorescence microscopic images showing the chemotactic effect of hydrogels on DC cells on the 12th day. Nuclei, blue; CD11c, Red. Scale bar, 50 μm. (C) Quantification of $CD11c^{+}$ cell populations in indicated groups. Data are expressed as mean ±S.E.M of three independent experiments. ns, no significance; ***, $p < 0.001$ by one-way ANOVA.

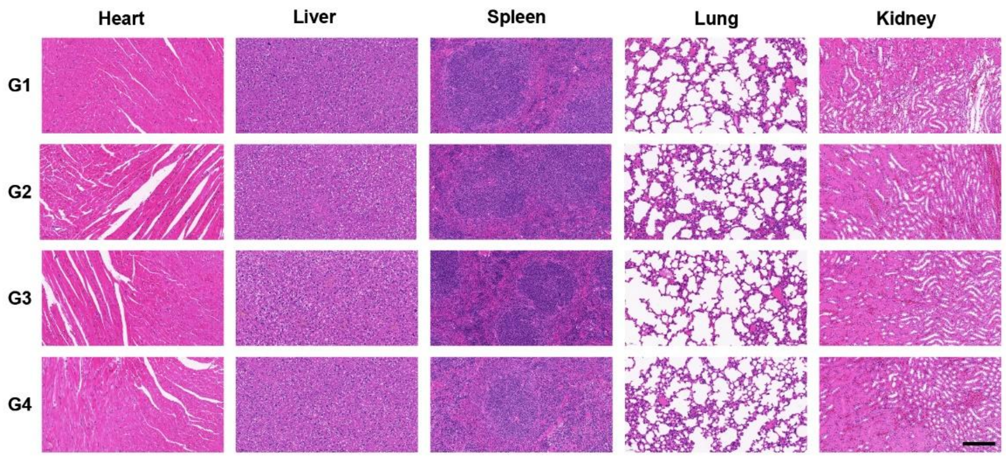

Figure S7. Histological analysis of the potential toxicity of the hydrogels. H&E staining images of heart, liver, spleen, lung and kidney collected on the 10th day from different groups. Scale bar, 150 μm.

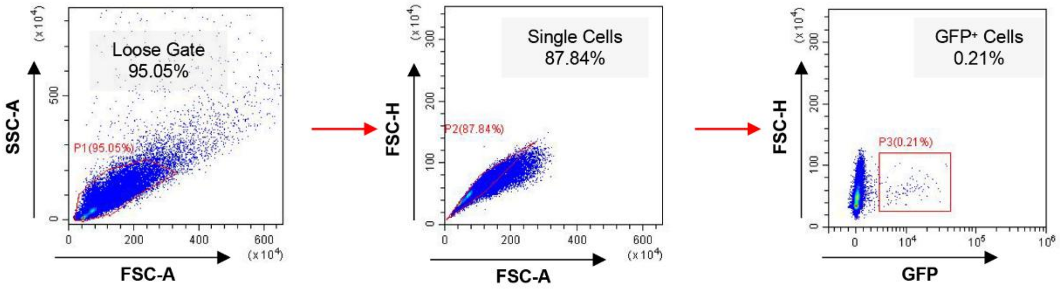

Figure S8. Flow cytometry gating scheme for analysis of circulating CT26.WT-GFP cells. The murine peripheral blood cells were isolated and subjected to FACS. Corresponding to Fig. 4J.

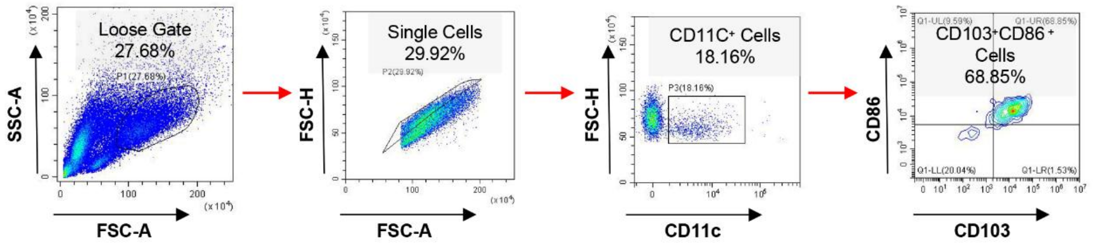

Figure S9. Flow cytometry gating scheme for analysis of mature cDC1 (CD11c$^+$CD103$^+$CD86$^+$ cells) in tumors. Isolated cells from tumors were stained and subjected to FACS. Corresponding to Fig. 5D.

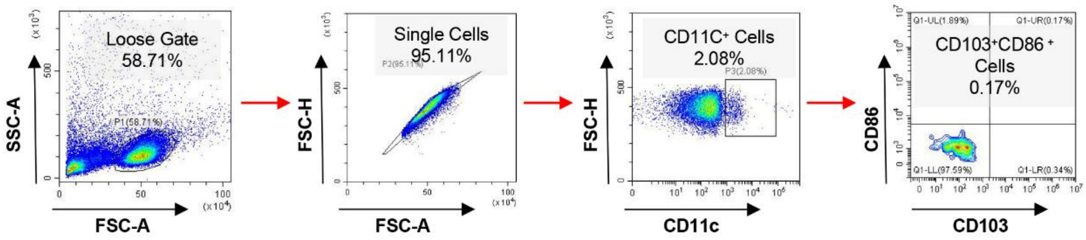

Figure S10. Flow cytometry gating scheme for analysis of mature cDC1 ($CD11c^{+}CD103^{+}CD86^{+}$ cells) in TdLN. Isolated cells from tumor-draining lymph nodes were stained and subjected to FACS. Corresponding to Fig. 5F.

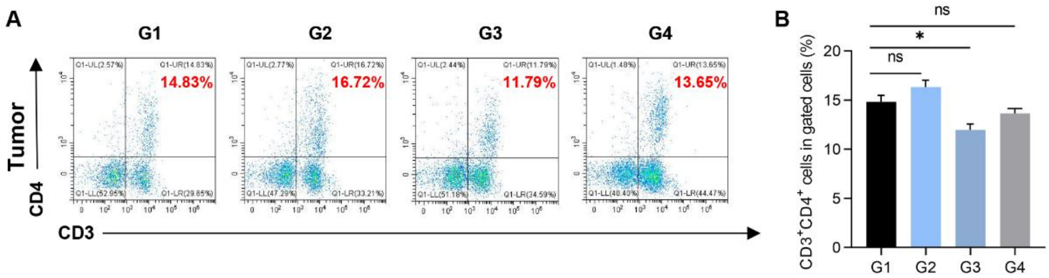

Figure S11. The effects of CCL21a/Exo$^{GM\text{-}CSF+Ce6}$@nanoGel/US on CD3$^+$CD4$^+$ population in CT26.WT tumor. (A) Representative flow cytometric analysis of CD3$^+$CD4$^+$ T cells in tumors from mice with indicated treatments. (B) Statistical analysis of Panel A. Data are expressed as mean ±S.E.M. n=6. ns, no significance; *, $p < 0.05$ by one-way ANOVA.

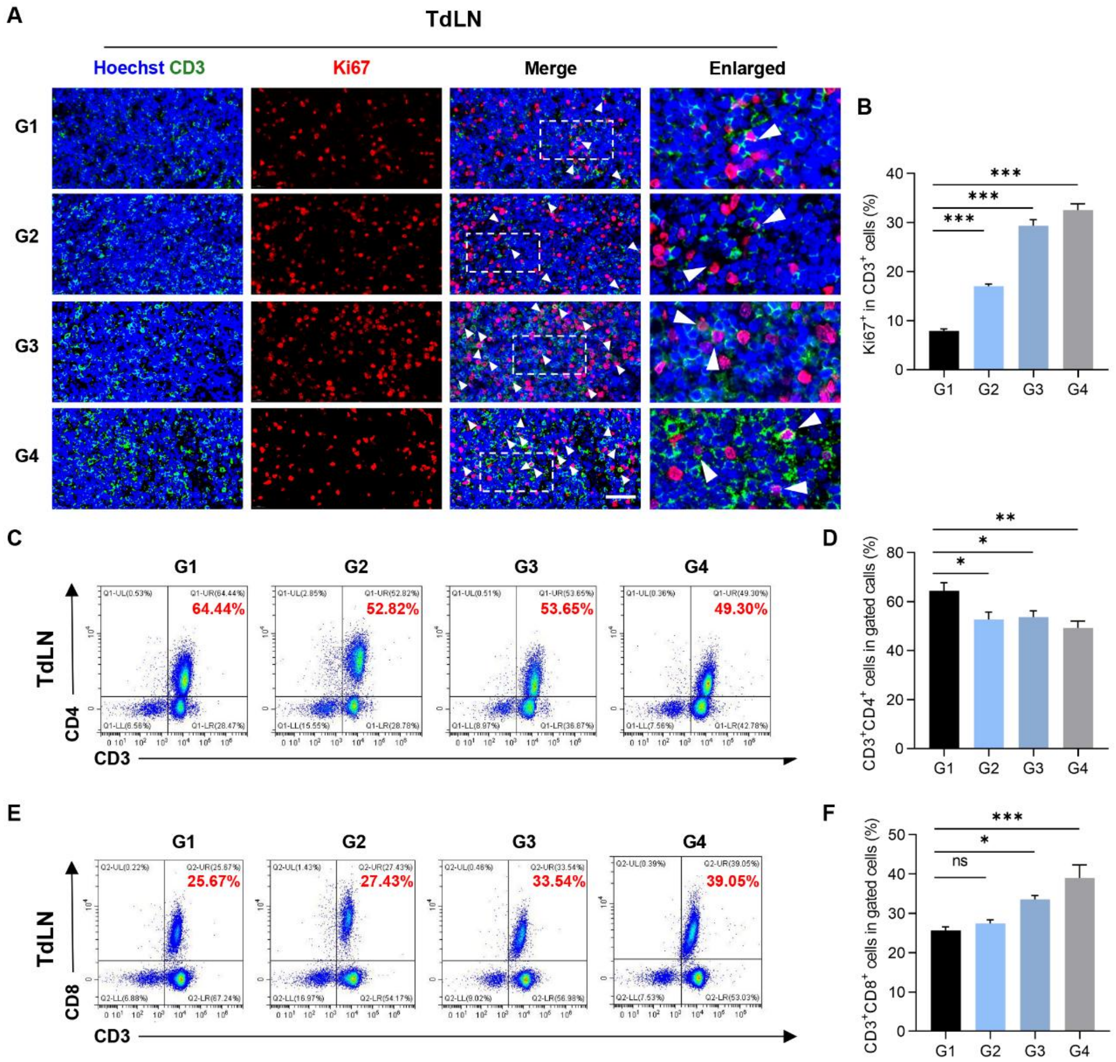

Figure S12. CCL21a/Exo$^{GM\text{-}CSF+Ce6}$@nanoGel/US promotes T cell priming in TdLN in CT26.WT mouse model. (A) Representative images of immunostaining of CD3 (green) and Ki67 (red) in tumor-draining lymph nodes from mice with indicated treatments. Scale bar, 50 μm. (B) Proportions of $Ki67^+$ cells in $CD3^+$ cells. Data are expressed as mean ±S.E.M. n=6. ***, $p<0.001$ by one-way ANOVA. (C) Representative flow cytometric analysis of $CD3^+CD4^+$ cells in tumor-draining lymph nodes from mice with indicated treatments. (D) Statistical analysis of Panel C. Data are expressed as mean ± S.E.M. n=6. *, $p < 0.05$; **, $p < 0.01$ by one-way ANOVA. (E) Representative flow cytometric analysis of CTL ($CD3^+CD8^+$ cells) in tumor-draining lymph nodes from mice with indicated treatments. (F) Statistical analysis of Panel E. Data are expressed as mean ± S.E.M. n=6. ns, no significance; *, $p<0.05$; ***, $p<0.001$ by one-way ANOVA.

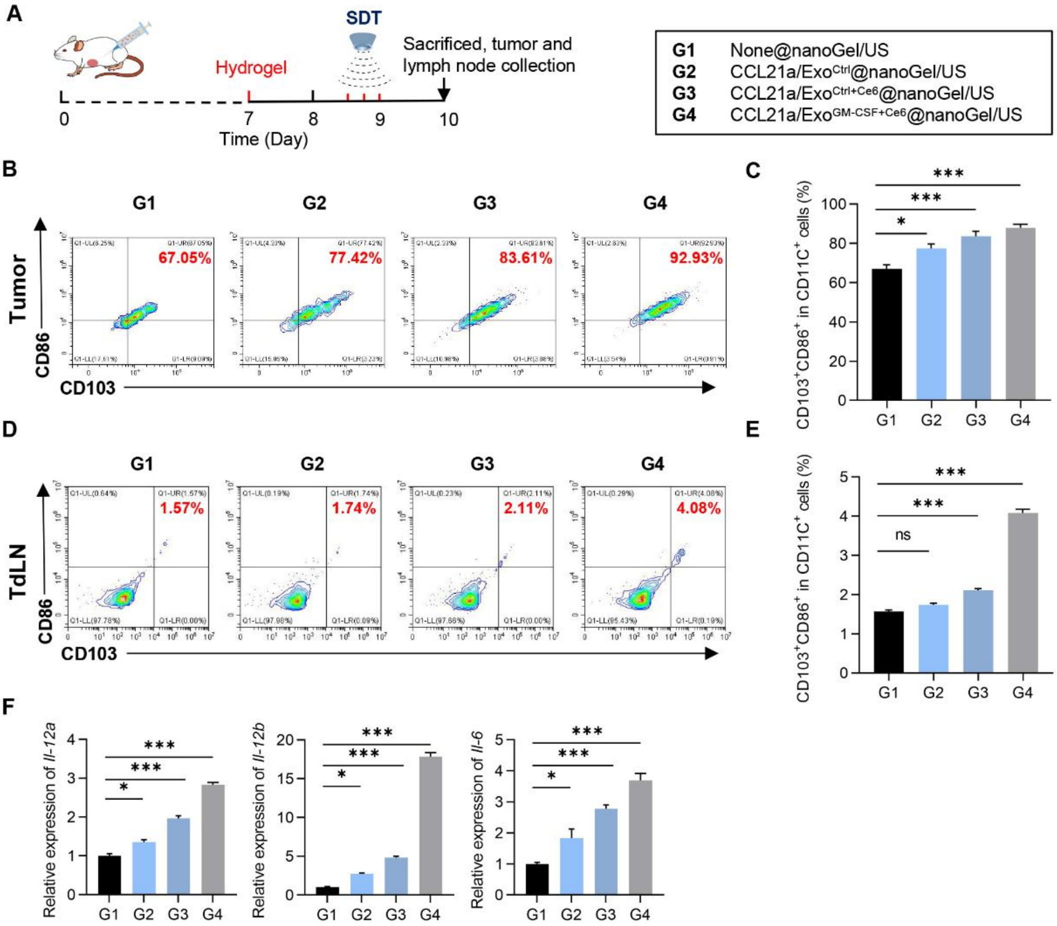

Figure S13. CCL21a/Exo$^{GM\text{-}CSF+Ce6}$@nanoGel/US promotes dendritic cell maturation in 4T1 breast cancer model. (A) Schematic representation of the experiment. 4T1 breast tumor bearing mice were injected with indicated hydrogels beside the tumor on the 7th day. Ultrasound irradiation was performed every 4 hours for three times on day 8. The mice were sacrificed and tumors and tumor-draining lymph nodes were harvested for further analysis. (B) Representative flow cytometric analysis of mature cDC1 ($CD103^{+}CD86^{+}$ cells) in tumors from mice with indicated treatments. (C) Statistical analysis of Panel D. Data are expressed as mean ± S.E.M. n=6. ns, no significance; *, p<0.05; **, p<0.01 by one-way ANOVA. (D) Representative flow cytometric analysis of mature cDC1 ($CD103^{+}CD86^{+}$ cells) in tumor-draining lymph nodes from mice with indicated treatments. (E) Statistical analysis of Panel F. Data are expressed as mean ± S.E.M. n=6. ns, no significance; ***, p<0.001 by one-way ANOVA. (F) qPCR analysis of the expression of *Il-12a*, *Il-12b* and *Il-6* in tumor-draining lymph nodes from mice with indicated treatments. Data are representative of three different experiments and expressed as mean ± S.E.M. *p < 0.05; ***p < 0.001 by one way ANOVA.

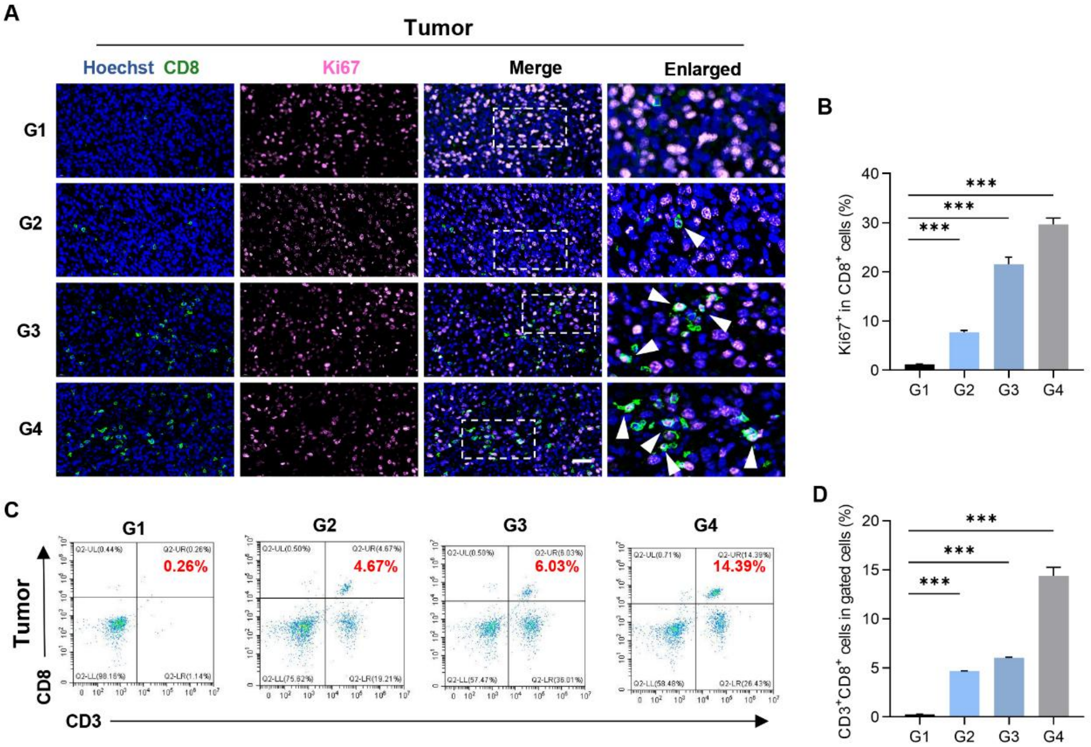

Figure S14. CCL21a/Exo$^{GM\text{-}CSF+Ce6}$@nanoGel/US activates T cell immune response in 4T1 breast cancer model. (A) Representative images of immunostaining of CD8 (green) and Ki67 (pink) in tumors from mice with indicated treatments. Scale bar, 50 μm. (B) Proportions of Ki67$^+$ cells in CD8$^+$ cells. Data are expressed as mean ±S.E.M. n=6. ***, p<0.001 by one-way ANOVA. (C) Representative flow cytometric analysis of CTL (CD3$^+$CD8$^+$ cells) in tumors from mice with indicated treatments. (D) Statistical analysis of Panel C. Data are expressed as mean ± S.E.M. n=6. ***, p<0.001 by one-way ANOVA.

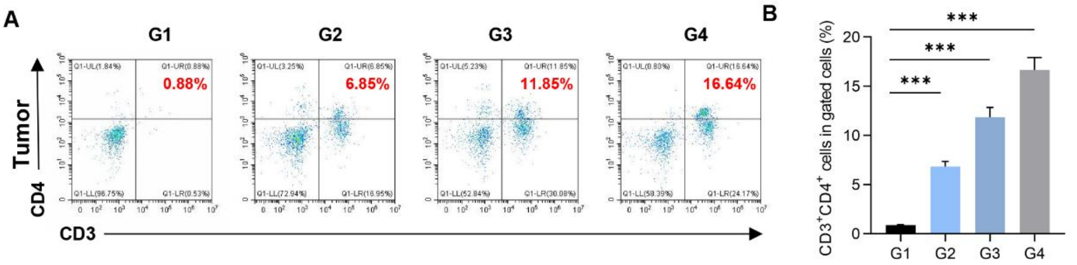

Figure S15. The effects of CCL21a/Exo$^{GM\text{-}CSF+Ce6}$@nanoGel/US on CD3$^+$CD4$^+$ cell infiltration in 4T1 tumor. (A) Representative flow cytometric analysis of CD3$^+$CD4$^+$ cells in tumors from mice with indicated treatments. (B) Statistical analysis of Panel A. Data are expressed as mean ± S.E.M. n=6. ***, $p < 0.001$ by one-way ANOVA.

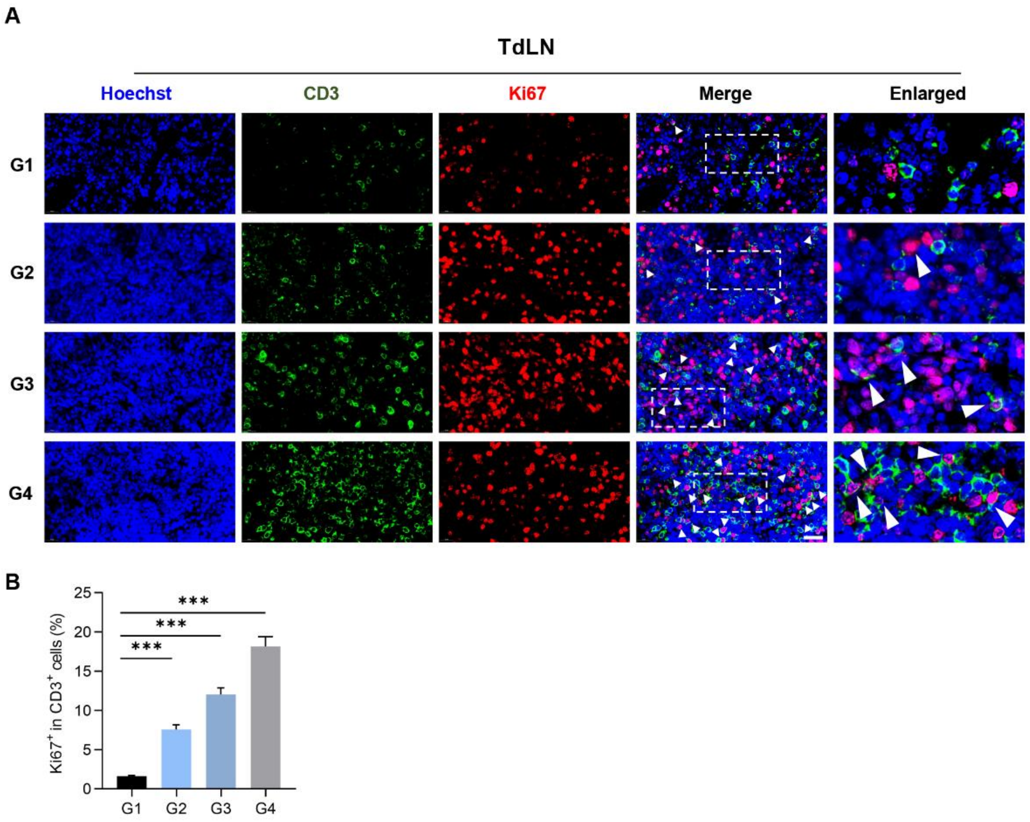

Figure S16. CCL21a/Exo$^{GM\text{-}CSF+Ce6}$@nanoGel/US promotes T cell proliferation in TdLN in 4T1 breast cancer model. (A) Representative images of immunostaining of CD3 (green) and Ki67 (red) in tumor-draining lymph nodes from mice with indicated treatments. Scale bar, 50 µm. (B) Proportions of Ki67$^+$ cells in CD3$^+$ cells. Data are expressed as mean ±S.E.M. n=6. ***, p<0.001 by one-way ANOVA.

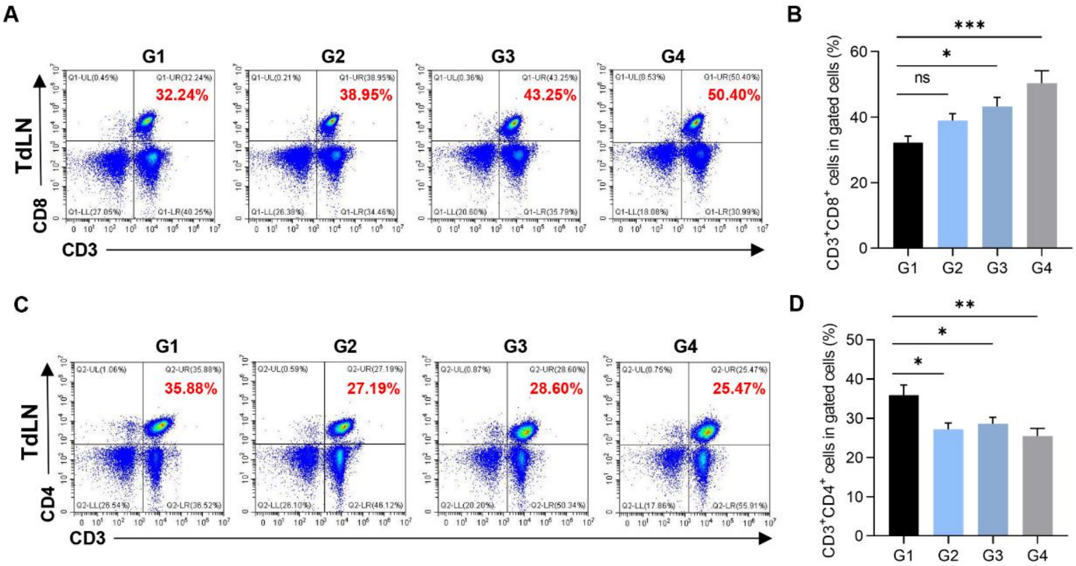

Figure S17. CCL21a/Exo$^{GM\text{-}CSF+Ce6}$@nanoGel/US promotes T cell priming in TdLN in 4T1 breast cancer model. (A) Representative flow cytometric analysis of CTL (CD3^{+}CD8^{+}cells) in tumor-draining lymph nodes from mice with indicated treatments. (B) Statistical analysis of Panel A. Data are expressed as mean ± S.E.M. n=6. ns, no significance; *, p<0.05; ***, p<0.001 by one-way ANOVA. (C) Representative flow cytometric analysis of CD3^{+}CD4^{+} cells in tumor-draining lymph nodes from mice with indicated treatments. (D) Statistical analysis of Panel C. Data are expressed as mean ± S.E.M. n=6. *, p < 0.05; **, p < 0.01 by one-way ANOVA.

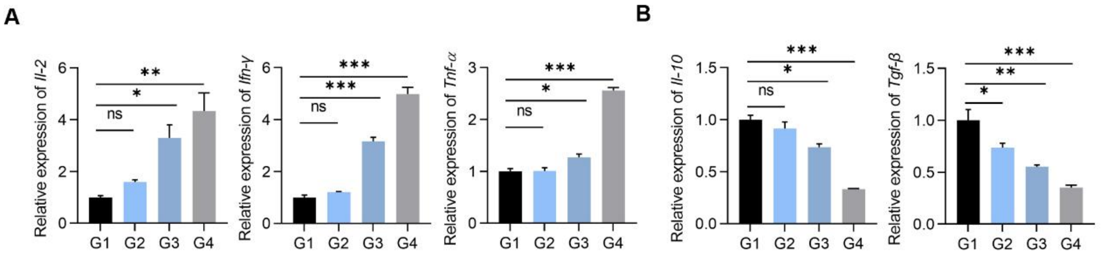

Figure S18. Effects of CCL21a/Exo$^{GM\text{-}CSF+Ce6}$@nanoGel/US on cytokine expression in 4T1 tumor. (A) qPCR analysis of the expression of *Il-2*, *Ifn-γ* and *Tnf-α* in tumors from mice with indicated treatments. Data are expressed as mean ± S.E.M. n=3 biological replicates. ns, no significance; *, p < 0.05; **, p < 0.01; ***, p < 0.001 by one way ANOVA. (B) qPCR analysis of the expression of *Il-10* and *Tgf-β* in tumors from mice with indicated treatments. Data are expressed as mean ± S.E.M. n=3 biological replicates. ns, no significance; *, p < 0.05; **, p < 0.01; ***, p < 0.001 by one way ANOVA.
